# Pulmonary toxicity assessment of polypropylene, polystyrene, and polyethylene microplastic fragments in mice

**DOI:** 10.1007/s43188-023-00224-x

**Published:** 2024-03-08

**Authors:** Isaac Kwabena Danso, Jong-Hwan Woo, Seung Hoon Baek, Kilsoo Kim, Kyuhong Lee

**Affiliations:** 1 Inhalation Toxicology Center for Airborne Risk Factor, Korea Institute of Toxicology, 30 Baehak1-Gil, Jeongeup, Jeollabuk-do 56212 Republic of Korea; 2 Department of Human and Environmental Toxicology, Korea National University of Science & Technology, Daejeon, 34113 Republic of Korea; 3 Biosafety Research Institute and Laboratory of Pathology, College of Veterinary Medicine, Jeonbuk National University, Jeollabuk do, Iksan-si, Republic of Korea; 4 Preclinical Research Center, Daegu-Gyeongbuk Medical Innovation Foundation, Daegu, 41061 Republic of Korea; 5 College of Veterinary Medicine, Kyungpook National University, 80 Daehakro, Buk-Gu, Daegu, 41566 Republic of Korea

**Keywords:** Microplastic, Fragment, TLR4, NF-ĸB, NLRP3 inflammasome

## Abstract

Polypropylene (PP), polystyrene (PS), and polyethylene (PE) plastics are commonly used in household items such as electronic housings, food packaging, bottles, bags, toys, and roofing membranes. The presence of inhalable microplastics in indoor air has become a topic of concern as many people spent extended periods of time indoors during the COVID-19 pandemic lockdown restrictions, however, the toxic effects on the respiratory system are not properly understood. We examined the toxicity of PP, PS, and PE microplastic fragments in the pulmonary system of C57BL/6 mice. For 14 days, mice were intratracheally instilled 5 mg/kg PP, PS, and PE daily. The number of inflammatory cells such as macrophages, neutrophils, and eosinophils in the bronchoalveolar lavage fluid (BALF) of PS-instilled mice was significantly higher than that in the vehicle control (VC). The levels of inflammatory cytokines and chemokines in BALF of PS-instilled mice increased compared to the VC. However, the inflammatory responses in PP- and PE-stimulated mice were not significantly different from those in the VC group. We observed elevated protein levels of toll-like receptor (TLR) 2 in the lung tissue of PP-instilled mice and TLR4 in the lung tissue of PS-instilled mice compared with those to the VC, while TLR1, TLR5, and TLR6 protein levels remained unchanged. Phosphorylation of nuclear factor kappa B (NF-κB) and IĸB-α increased significantly in PS-instilled mice compared with that in VC. Furthermore, Nucleotide‑binding oligomerization domain‑like receptor family pyrin domain‑containing 3 (NLRP3) inflammasome components including NLRP3, apoptosis-associated speck-like protein containing a caspase recruitment domain (ASC), and Caspase-1 in the lung tissue of PS-instilled mice increased compared with that in the VC, but not in PP- and PE-instilled mice. These results suggest that PS microplastic fragment stimulation induces pulmonary inflammation due to NF-ĸB and NLRP3 inflammasome activation by the TLR4 pathway.

**Supplementary Information:**

The online version contains supplementary material available at 10.1007/s43188-023-00224-x.

## Introduction

Microplastics have become ubiquitous in the environment owing to the increasing production and consumption of plastic products, coupled with inadequate disposal and slow biodegradation [1–3]. Microplastics exist primarily as purposefully manufactured micro-sized products, or secondary microplastics formed from the disintegration of plastic debris exposed to ultraviolet radiation, mechanical stress, and biological actions in the environment [4]. As a result, the occurrence of microplastics in the environment is diverse, consisting of a wide range of polymer types such as polypropylene (PP), polystyrene (PS), and polyethylene (PE), with different sizes and shapes including beads, fragments, and fibres [2, 5]. Microplastic pollution has been previously discussed as a marine environmental issue. However, in recent times, its occurrence in both indoor and outdoor air environments has been confirmed, with reports suggesting that inhalation is a more dominant route of exposure [6–9]. Although research on airborne microplastic exposure has primarily focused on outdoor environments, a limited number of studies have revealed that indoor microplastic concentrations are notably elevated. These elevated indoor microplastic levels may exert adverse effects on human health [10–12]. Furthermore, the COVID-19 pandemic and associated lockdown regulations have led to prolonged indoor periods for individuals, underscoring the significance of this study. Despite the potential risks involved, our understanding of the pulmonary toxicity of inhaled microplastics in humans remains limited.

In recent studies, the fragment and fibre shapes in atmospheric microplastics were observed to be the most predominant, and it has also been reported that humans are exposed to an average of 55,000 particles annually via the inhalation route [13–15]. In addition, microplastics of 12 polymer types including PP, PS, and PE were detected in the lung tissue of humans, with most of them being fragments (43%) and fibre (49%) [16]. Microplastic particles have also been observed in the sputum of patients with various respiratory diseases [17]. Previous studies have reported that some occupational workers with long-term exposure to microplastics developed pulmonary diseases including lung cancer and asthma [2, 3, 18–21]. Although some studies have highlighted the potential health risks associated with the inhalation of microplastics, our understanding of the toxicity mechanisms of different types of microplastics within the respiratory system remains limited.

Toll-like receptors (TLRs) are pattern recognition receptors (PRRs) that detect both exogenous pathogen-associated molecular patterns (PAMPs) and endogenous danger-associated molecular patterns (DAMPs), thereby triggering host defense responses and inflammations [22–24]. TLRs can activate multiple intracellular signals, including the nuclear factor κB (NF-κB) pathway, which plays a major role in regulating inflammatory responses [25]. Recent research has indicated that NF-κB serves as an integral component of the initiation signal required for stimulation of the NLRP3 inflammasome, which subsequently triggers the activation of Caspase-1 and consequent release of interleukin (IL)-1β [26, 27]. TLR-mediated inflammation is implicated in pulmonary diseases including asthma, pulmonary fibrosis, and chronic obstructive pulmonary disease (COPD) [28–32]. Recent research has reported that air pollution agents such as particulate matter, activated TLR2- and TLR4-mediated lung inflammations [33–35], however, the mechanism of pathogenesis for lung inflammation due to microplastic exposure is still unclear.

This study investigated inflammatory responses including inflammatory cytokine and chemokine levels, cellular changes, and histopathological analysis of bronchoalveolar lavage fluid (BALF) and lung tissue of PP-, PS-, and PE-instilled mice. Additionally, we examined the mechanism of toxicity of TLR-mediated lung inflammation in the lung tissues of microplastic-instilled mice.

## Materials and methods

### PP, PS, and PE microplastic fragments

PP beads (PropylTex® 50, Micro Powders Inc., New York, NY, USA) and PE beads (5 mm) were purchased to prepare PP and PE microplastic fragments of size approximately <20 µm. PP and PE beads were frozen at −78 °C and subsequently ground into a powder using a blade-shaped homogenizer, a process lasting approximately 4 h. PP and PE beads were frozen at -78 °C and subsequently homogenized to a powder using a blade-shaped device; this process lasted approximately 4 h. The resulting powder was passed through a 50-µm mesh and washed five times with ethanol. The samples were then dried at 50 °C for 48 h. To produce PP and PE microplastics with particle sizes of approximately <20 µm, microplastics were dispersed in ethanol and subjected to high-pressure homogenization (four passes at 600 bar). Subsequently, the resulting particles were passed through 15-µm mesh filters, subjected to five ethanol washes, and then dried for 48 h at 50 °C. PS microplastic fragments were supplied by the Korea Testing and Research Institute. To prepare microplastic fragments for experimentation, PP, PS, and PE microplastics were dispersed in a solution consisting of 1% DMSO in saline. The resulting solution was sonicated in a water bath for 30 min. Field-Emission Scanning Electron Microscopy (FE-SEM) (S-4800, Hitachi, Japan) analysis was performed to determine the shape and size of the three microplastic fragments. Zeta potentials (ELSZ-2000, Otsuka, Japan) were measured in triplicate to determine their respective surface charges.

### Animals and experimental design

Male C57BL/6 mice of seven weeks old were procured from Orient Bio Inc. (Seongnam, Korea) and housed in a controlled environment at constant temperature and relative humidity of 22 ± 3 °C and 50 ± 20% respectively, and a 12 h light/dark cycle. Throughout the experiment, mice were provided with standard experimental rodent pellets (PMI Nutrition International, Richmond, IN, USA) and UV-sterilized and filtered tap water ad libitum. Experimental procedures were carried out with approval from the Institutional Animal Care and Use Committee at the Korea Institute of Toxicology (IACUC #2108–0023). In the PP, PS, and PE experimental groups, mice were subjected to intratracheal instillation of 5 mg/kg of PP, PS, and PE suspended in a 50 μl saline solution over the 2 weeks, employing an automatic instillator [36]. Similarly, mice in the vehicle control (VC) group were also intratracheally instilled with saline. Mice of all groups were sacrificed on day 15.

### Bronchoalveolar lavage fluid (BALF) analysis

At 24 h after the administration of the three microplastics (PP, PS, and PE), the mice were anesthetized using isoflurane and euthanized by exsanguination. The left lung was ligated and the trachea cannulated. Subsequently, the right lung was lavaged three times, with 0.7 mL of phosphate-buffered saline (PBS). Total cells of the collected BALF were counted with the help of a NucleoCounter (NC-250; ChemoMetec, Gydevang, Denmark). BALF cell smears were prepared for differential cell counts using Cytospin (Thermo Fisher Scientific) and stained with Diff-Quik solution (Dade Diagnostics, Aguada, Puerto, USA). A total of 200 cells were counted per slide.

### Histopathological analysis

The left lung tissue of mice were fixed in 10% neutral-buffered formalin. The tissue specimens were dehydrated and embedded in paraffin. Subsequently, Sects. (4-μm-thick) were stained with hematoxylin and eosin (H&E). All fields per section from each animal were analyzed using a Leica DM2500 microscope (Leica Instruments, Wetzlar, Germany) at 200 × and 400 × magnifications.

### Inflammatory cytokine and chemokine levels in BALF

The levels of IL-1β, IL-6, monocyte chemoattractant protein-1 (MCP-1), macrophage inflammatory protein (MIP)-1α, MIP-2, and C-X-C motif chemokine ligand 1 (CXCL1/KC) in BALF were measured using commercial ELISA kits (R&D System) according to the manufacturer’s instructions.

### Preparation of protein extract and western blot analysis

Lung tissues were homogenized using RIPA buffer (Thermo Fisher Scientific) supplemented with a protease and phosphatase inhibitor cocktail, following the manufacturer's instructions. Protein concentrations were quantified using the Bradford reagent (Bio-Rad). The samples were subsequently loaded and separated using sodium dodecyl sulfate–polyacrylamide gel electrophoresis at 90 V for 120 min. Following electrophoresis, the samples were transferred by the wet method unto polyvinylidene difluoride membranes (Merck Millipore) at a current of 250 mA for 60 min. After blocking non-specific sites with 5% non-fat dry milk in 0.1% Tween 20 in Tris-buffered saline (TBS-T) for 1 h, the membrane was incubated overnight at 4 ℃ with TLR1 (Abeomics, San Diego, USA), TLR2 (Abcam, Cambridge, UK), TLR4 (Invitrogen, Massachusetts, USA), TLR5 (Abcam, Cambridge, UK), TLR6 (Boster Bio, Pleasanton, USA), NF-κB (Cell Signaling, Massachusetts, USA), p-NF-κB (Cell Signaling, Massachusetts, USA), IκB (Cell Signaling, Massachusetts, USA), p-IκB (Cell Signaling, Massachusetts, USA), NLRP3 (AdipoGen Life Sciences, Inc. Liestal, Switzerland), ASC (AdipoGen Life Sciences, Inc. Liestal, Switzerland), Caspase-1 (AdipoGen Life Sciences, Inc. Liestal, Switzerland), and β-actin (Santa Cruz Biotechnology, Dallas, TX, USA). Horseradish peroxidase-linked anti-rabbit IgG (Cell Signaling, Massachusetts, USA) and anti-mouse IgG (Cell Signaling, Massachusetts, USA) were used to detect antibody binding and with the help of iBright CL 1000 imaging system (Thermo Fisher Scientific), bands were visualized after treatment with the ECL reagent (Thermo Fisher Scientific). The results of the densitometric analysis were expressed as the relative ratio of the target protein to the reference protein. The relative ratio of the target protein to the control was arbitrarily denoted as 1.

### Statistical analysis

All statistical analyses were performed using GraphPad InStat v. 3.0 (GraphPad Software, Inc., La Jolla, CA, USA). Statistical comparisons between more than two groups were performed using one-way analysis of variance (ANOVA) followed by Dunnett’s multiple comparison test, and statistical comparisons between two groups were conducted using Student’s t-test. Data are presented as the mean ± SD. A value of *p* <0.05 was considered to indicate statistically significant results.

## Results

### Characterization of PP, PS, and PE microplastic fragments

**Fig. 1:**
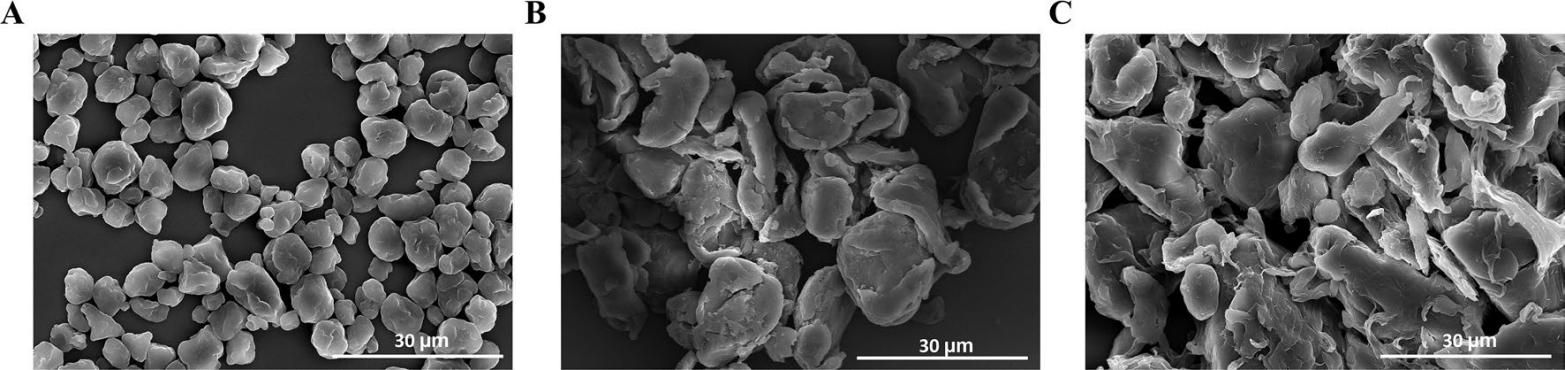
FE-SEM images of microplastic fragments **a** PP, **b** PS, and **c** PE. Scale bar 30 μm

The microplastic particles generally appeared as irregular fragment shapes. The results revealed that the microplastic fragments had a diameter of 6.40 ± 1.48 µm for PP (Fig. 1a). Additionally, the average diameters of PS and PE microplastics were 17.53 ± 2.11 µm and 21.27 ± 6.07 µm, respectively (Fig. 1b-c). The zeta potential of PP, PS, and PE fragments was −8.28 ± 1.37, −38.93 ± 4.49, and −5.71 ± 1.10 mV, respectively (Table 1).

**Table 1:** Surface charges of PP, PS, and PE microplastic fragments

	Microplastic Fragments		
	PP	PS	PE
Zeta Potentials (mV)	−8.28 ± 1.37	−38.93 ± 4.49	−5.71 ± 1.10

### Inflammatory response in PP, PS, and PE-stimulated mice

**Fig. 2:**
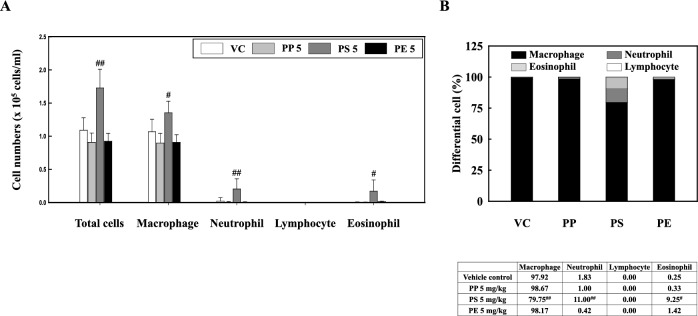
**a** Cellular changes in the BALF of mice stimulated with three microplastics (PP, PS, and PE). **b** Total and differential cells in BALF. Data are presented as mean ± SD (*n* = 6–8 per group). ^#^*p* ≤ 0.05; ^##^*p* ≤ 0.01 vs. VC

**Fig. 3:**
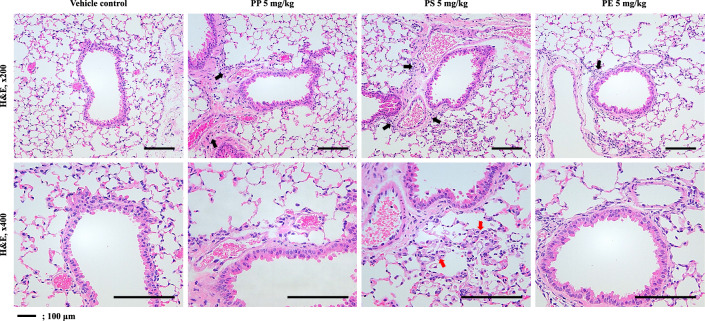
Representative H&E-stained section of lung tissue. Black and red arrows indicate inflammatory cell infiltration and macrophage increased. Scale bar 100 μm

**Fig. 4:**
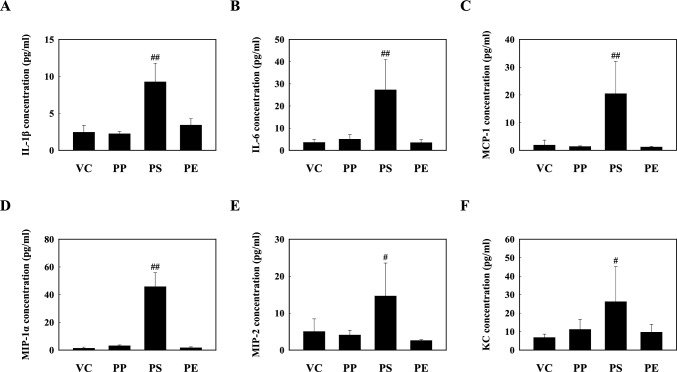
Inflammatory cytokines & chemokines levels, including **a** IL-1β, **b** IL-6, **c** MCP-1, **d** MIP-1α, **e** MIP-2, and **f** KC in the BALF of mice instilled with PP, PS, and PE microplastic fragments. Data are presented as mean ± SD (*n* = 6–8 per group). ^#^*p* ≤ 0.05; ^##^*p* ≤ 0.01 vs. VC

We examined the inflammatory response to PP, PS, and PE microplastic fragment stimulation. Our results showed that total cells, macrophages, neutrophils, and eosinophils were significantly increased in the PS stimulation mice compared to those in the VC (Fig. 2a). The percentage of macrophages in the BALF of PS-instilled mice decreased significantly to 79.75% compared to that in VC. However, the neutrophil percentage (11.00%) and eosinophil percentage (9.25%) were significantly higher than those in VC (Fig. 2b). The inflammatory cellular changes in the BALF of PP- and PE-instilled mice were not significantly different from those in the VC (Fig. 2). Histopathological analysis of the lung tissues of PP-, PS-, and PE-instilled mice showed inflammatory cell infiltration. In addition, the PS-stimulated mice showed increased macrophage infiltration (Fig. 3). Furthermore, the levels of inflammatory cytokines such as IL-1β and IL-6 in the BALF of PS-instilled mice were higher than those in the VC, but not in PP- and PE-instilled mice (Fig. 4a, b). Our results showed that the levels of inflammatory chemokines including MCP-1, MIP-1α, MIP-2, and KC increased in the 5 mg/kg PS-instilled mice compared to those in the VC; however, the inflammatory chemokine levels in PP- and PE-instilled mice remained significantly unchanged (Fig. 4c–f).

### Microplastic fragment stimulation induces TLRs activation

**Fig. 5:**
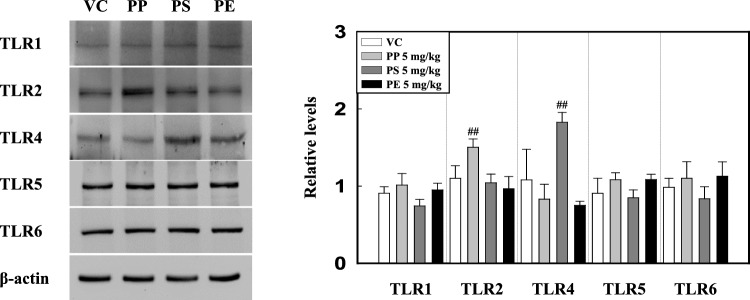
Representative western blotting analysis and relative density of TLRs 1, 2, 4, 5, and 6 in the lung tissue of PP-, PS-, and PE-instilled mice. Data were normalized against β-actin. Data are presented as mean ± SD (*n* = 6–8 per group). ^##^*p* ≤ 0.01 vs. VC

TLRs are a group of proteins involved in the early stages of the host defense against invading pathogens, which is an important factor in the regulation of inflammatory response [22–25]. We investigated the protein levels of TLRs in the lung tissue of microplastic-instilled mice. Our results showed that PS-instilled mice had significantly increased expression levels of TLR4 as compared to the VC group. However, the levels of TLR1, 2, 5, and 6 in PS-instilled mice remained unchanged compared to those in the VC group (Fig. 5). Interestingly, PP-instilled mice showed a significant increase in the expression levels of TLR2 as compared to the VC group. The expression levels of TLR1, 4, 5, and 6 did not significantly increase in PP-instilled mice (Fig. 5). In PE-instilled mice, the protein levels of all TLRs were not significantly different from those in VC (Fig. 5).

### PS microplastic fragments stimulation activates NLRP3 Inflammasome through NF-κB signaling pathway

**Fig. 6:**
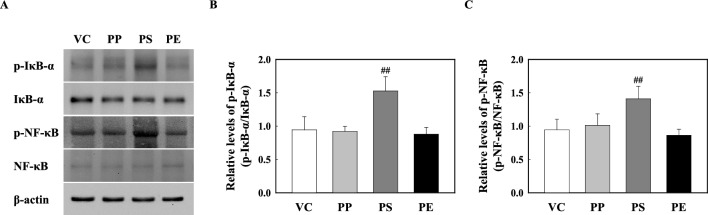
**a** Representative western blot analysis of p-IκB-α, IκB-α, p-NF-κB, and NF-κB in the lung tissue of PP-, PS-, and PE-instilled mice. **b** Relative density analysis of p-IκB-α levels. Data were normalized against IκB-α. **c** Relative density analysis of p-NF-κB levels. Data were normalized against NF-κB. Data are presented as mean ± SD (*n* = 6–8 per group). ^##^*p* ≤ 0.01 vs. VC

**Fig. 7:**
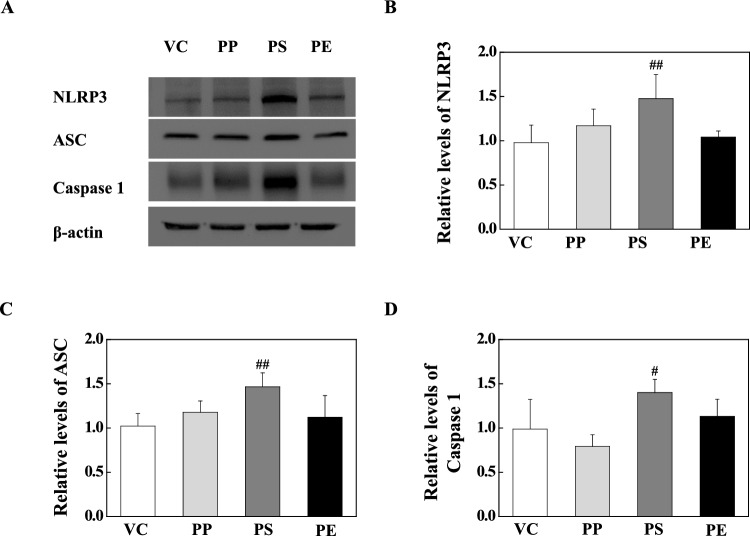
**a** Representative western blot analysis of NLRP3, ASC, and Caspase-1 in lung tissue of PP-, PS-, and PE-instilled mice. **b** Relative density analysis of NLRP3 levels. **c** Relative density analysis of ASC levels. **d** Relative density analysis of Caspase-1 levels. Data were normalized against β-actin. Data are presented as mean ± SD (*n* = 6–8 per group). ^#^*p* ≤ 0.05; ^##^*p* ≤ 0.01 vs. VC

Our results showed that the p-IκB-α protein levels in the lung tissue of PS-instilled mice were significantly increased compared to those in VC. Additionally, the p-NF-κB protein levels in the lung tissue of PS-stimulated mice increased compared to those in the VC (Fig. 6). However, the protein levels of IκB-α and NF-κB phosphorylation in the lung tissues of PP- and PE-instilled mice remained unchanged (Fig. 6). Furthermore, we observed the protein levels of NLRP3 inflammasome components such as NLRP3, ASC, and Caspase-1. Our results showed that NLRP3, ASC, and Caspase-1 expression were significantly increased in the lung tissue of PS-stimulated mice compared to that in the VC, while PP- and PE-instilled mice did not show significant changes (Fig. 7).

## Discussion

We investigated the molecular mechanism of the pulmonary toxicity response to PP, PS, and PE microplastic fragment stimulation in mice. Our results showed the inflammatory response including inflammatory cells, cytokines, and chemokines in BALF of PS intratracheal instillation mice increased compared to the VC. Histopathological analysis of the lung tissue of PS-instilled mice revealed lung injury such as inflammatory infiltration in the perivascular/peribronchial region. The PS fragments stimulation significantly increased the protein levels of TLR4 in the lung tissue with respect to the VC, but not protein levels of TLR1, 2, 5, and 6. The protein levels of IκB-α and NF-κB phosphorylation in the lung tissue of PS-treated mice were significantly increased compared to those in VC. PS stimulation led to a significant increase in NLRP3 inflammasome components including NLRP3, ASC, and Caspase-1. These results suggest that PS microplastic fragments may contribute to NF-κB and NLRP3-mediated inflammation via the TLR4 signaling pathway in the respiratory system.

The detrimental effects of airborne microplastics on the pulmonary system have been rarely reported. Although in vitro and in vivo studies have demonstrated threats, the variations in the characteristics of environmental microplastics require wider toxicity investigations for a better understanding. Previous studies have explored the effects of microplastic exposure on different polymers in the pulmonary system. Specifically, 6.25 mg/kg of PS microplastic intratracheal instillation in mice has been observed to induce pulmonary inflammation [37]. In another study, the administration of PS microplastic via intranasal instillation daily at a dose of 40 mg/kg for 21 days resulted in a significant increase in the levels of inflammatory cytokines in the lungs of mice [38]. PE microplastics have also been reported to exert inflammatory effects on mouse lungs at concentrations ranging between 500 to 2,000 mg/kg after 28 days of exposure via oral administration. In this study, the no-observed-adverse-effect level (NOAEL) was estimated to be less than 1,000 mg/kg in male mice and <500 mg/kg in female mice [39]. An evaluation of the toxicity of polypropylene fragments through oral administration in mice showed that the NOAEL for PP microplastics was greater than 2,000 mg/kg [40]. Nonetheless, it is worth noting that intratracheal instillation of PP microplastic in mice induces inflammation at a dose of 2.5 mg/kg, as demonstrated in a previous study [41]. This discrepancy in microplastic doses among toxicity assessments highlights the variations in our understanding of their effects. Furthermore, the environmental concentrations of microplastics tend to vary depending on the catchment area. A recent study in Shanghai estimated that daily human exposure to inhalable indoor aerosols is approximately 704 ± 254 microplastic items with approximately 526 ± 203 microplastic items deposited in the pulmonary airway [42]. In other parts, microplastic concentrations range between 230 ± 94 and 358 ± 132 items/m^3^, mostly as fragments and fibres [43]. Generally, adult humans inhale approximately 6.5–8.97 μg/kg microplastics daily. However, this rate can be significantly higher in infants, ranging from 3 to 50 times the adult levels [44]. Therefore, we observed the pulmonary toxic effects of 5 mg/kg (daily concentration) in PP-, PS-, and PE-instilled mice.

Previous studies have revealed various physiological dysfunctions caused by microplastic exposure in vivo and in vitro [38, 41, 45, 46]. These effects depend on factors such as size and shape [3]. Using FE-SEM imaging, we confirmed the shapes and sizes of PP, PS, and PE microplastic fragments. PP, PS, and PE particles were observed to have relatively irregular morphologies with average sizes of about 6.40 ± 1.48 µm, 17.53 ± 2.11 µm, and 21.27 ± 6.07 µm respectively (Fig. 1). Studies have reported that microplastic exposure induces toxicity in various biological systems in a size-dependent manner [5, 47]. However, in this study, PS-instilled mice showed significantly higher toxicity responses, including an increase in cellular recruitment and inflammatory cytokine and chemokine levels than PP- and PE-instilled mice (Figs. 2 and 4), although PP microplastic fragments were the smallest. We hypothesized that a combination of other additional properties such as surface charges might account for these responses. Owing to their elevated surface-to-volume ratio, the surface charges of microplastics have been reported to play a crucial role in influencing their functions and interactions within biological systems, potentially resulting in adverse effects [48]. Zeta potential measurements provide information about particle charges and dispersion stability, with absolute values above 30 mV indicating low aggregation that leads to good homogenization following exposure [49, 50]. Although the role of surface charge in microplastic toxicity has been sparsely reported, inhalation of negatively charged PS microplastic (zeta potential of—35.98 ± 0.26 mV) reportedly induced an influx of leukocytes and inflammatory cytokine expression in BALF and lung tissues of mice [51]. In accordance with the findings of Shao et al., polymeric particles that share similar charges, irrespective of whether negative or positive, tend to exhibit increased cytotoxicity and enhanced affinity for cells as their charge values increase [52]. Similarly, the mean zeta potentials recorded for PP, PS, and PE fragments in this study were −8.28 ± 1.37, −38.93 ± 4.49, and −5.71 ± 1.10 mV, respectively (Table 1), following a toxicity trend of PE (−5.71 ± 1.10 mV) < PP (−8.28 ± 1.37 mV) < PS (−38.93 ± 4.49 mV). The interplay of these properties may contribute to the inflammatory response for microplastic exposure. Hence, further studies are imperative to gain a comprehensive understanding of the precise roles and mechanisms through which these physicochemical characteristics influence both short-term and long-term exposure effects.

Immune cells play an essential role in homeostasis maintenance in the lung by recognizing and eliminating inhaled foreign substances; however, excessive infiltration of inflammatory cells may cause lung injury [53–55]. Our results showed that PS stimulation significantly increased the number of inflammatory cells including macrophages, neutrophils, and eosinophils, in the BALF of mice (Fig. 2). In addition, the levels of inflammatory chemokines, including MCP-1, MIP-1α, MIP-2, and KC in the BALF of PS-instilled mice significantly increased compared to those in the VC (Fig. 4c-f). Previous studies have reported that airborne particles such as particulate matter increased the number of macrophages, neutrophils, and eosinophils in the BALF of mice and the levels of inflammatory chemokines, including MCP-1 were increased compared to those in the VC [56]. MCP-1 is a key chemokine involved in the migration and infiltration of monocytes/macrophages and also plays a role in the recruitment of eosinophils to acute and chronic inflammatory sites [57, 58]. Recent studies reported that diesel exhaust particle stimulation increased the number of neutrophils and the levels of inflammatory cytokines and chemokines such as IL-6, MCP-1, MIP-2, and KC in the lung tissue of mice [59]. The chemokines MIP-2 and KC are linked to the influx of neutrophils in the rodent lung [60], both of which have been implicated in the inflammatory process [61, 62]. These results suggest that PS stimulation causes cellular recruitment and inflammatory cytokine and chemokine release, which might lead to pulmonary inflammation in the respiratory system.

TLRs are essential components of the innate immune system against invading pathogens through their recognition of molecular patterns and subsequent initiation of the inflammatory response [33–35]. Recent studies have reported that most air pollution agents, such as particulate matter, induce inflammations through TLR2- and TLR4-mediated signaling, which is detected by the endogenous DAMP ligands released by tissue injury [63–65]. Recent studies have demonstrated differences in the functions of TLRs. TLR1, TLR2, and TLR6 require heterodimer formation such as TLR1/TLR2 and TLR2/TLR6 for the activation of inflammatory responses including IL-1β secretion, whereas TLR4 activates the inflammatory responses as a homodimer [66–68]. TLRs initiate signaling pathways that result in the nuclear translocation of NF-κB and NLRP3 inflammasome activation, which play an essential role in the pathogenesis of lung inflammation via cytokine release and mediators [63]. We examined the protein levels of TLRs in the lung tissues of PP, PS, and PE microplastic fragment-stimulated mice. The TLR2 level in the lung tissue of PP-stimulated mice significantly increased compared with that in the VC, but the levels of TLR1, TLR4, TLR5, and TLR6 did not increase (Fig. 5). PP-instilled mice showed no change in protein levels of p-IκB-α, p-NF-κB, and NLRP3 inflammasome components compared with those in the VC (Fig. 6 and 7). These results show that PP stimulation increases the TLR2 level; however, the absence of TLR1 and TLR6 might result in no heterodimer formation. On the other hand, PS stimulation significantly increased the protein level of TLR4 (Fig. 5). The protein levels of p-IκB-alpha and p-NF-κB in the lung tissues of PS-instilled mice significantly increased compared to the VC (Fig. 6). In addition, a significant increase in the protein levels of NLRP3 inflammasome components including NLRP3, ASC, and Caspase-1 of lung tissue in PS-stimulated mice as compared to the VC (Fig. 7). These results showed that PS microplastic fragment stimulation may induce pulmonary inflammation linked to NLRP3 and NF-κB through the TLR4 signaling pathway.

### Supplementary Information

Below is the link to the electronic supplementary material.

Supplementary file1 (PDF 326 KB)

Supplementary file2 (PDF 1728 KB)

Supplementary file3 (PDF 298 KB)

## Data Availability

All datasets generated during the current study are available from the corresponding author on request.
