# Supplementary material for: Pulmonary toxicity assessment of polypropylene, polystyrene, and polyethylene microplastic fragments in mice
Source: Toxicol Res. 2024 Mar 8;40(2):313–23. doi: 10.1007/s43188-023-00224-x (PMC10959865; doi:10.1007/s43188-023-00224-x)
Supplement: Supplementary file 1 — Supplementary file1 (PDF 326 KB) [file 43188_2023_224_MOESM1_ESM.pdf]

중앙대학교 대학원
카피킬러캠퍼스 표절 검사
결과 확인서

확 인
성 명
서 명

아이디	2020220141	표절률	11%
소속	대학원		
성명	자필로 기재하세요		

검사번호	00250062289	검사일자	2023.12.14 18:54
발급형태	▫ 기본보기 ▫ 요약보기 ■ 상세보기	발급일자	2023.12.14 18:55
검사명	학술논문 검사		
문서명	Manuscript_Toxicological research 12.14_final.docx		
비고			

비교범위	[현재첨부문서] [카피킬러 DB]
검사설정	표절기준 [6 어절], 인용/출처 표시문장 [제외], 법령/경전 포함문장 [제외], 목차/참고문헌 [제외]

검토 의견	

Copyright ⓒ MUHAYU All Rights reserved.

분석 정보

표절률	전체문장	동일문장	의심문장	인용/출처	법령/경전
11%	164	1	29	2	0

비교 문서 정보

번호	표절률	출처정보	비고
1	6%	[카피킬러 DB] www.ncbi.nlm.nih.gov<br>- 파일명 : Polypropylene nanoplastic exposure leads to lung inflammation through p38-mediated NF-κB pathway due to mitochondrial damage - PMC	
2	1%	[카피킬러 DB] www.ncbi.nlm.nih.gov<br>- 파일명 : Oral administration of Jinan Red Ginseng and licorice extract ...<br>- 저자 : Daram Yang, Hyuneui Jeong, Seung-Mi Hwang, Jong-Won Kim, Hee-Won Moon, Ye-Eun Lee, Hyo-Bin Oh, Chung-berm Park, Bumseok Kim<br>- 발행 : January 2022	
3	1%	[카피킬러 DB] Copykiller<br>- 파일명 : Comparative toxicity of polyhexamethylene guanidine phosphate in three strains of rats<br>- 저자 : Woo Jong-Hwan Kim Ki Cheon Kim Hyeon-Young Kim In-Hyeon Kim Sung-Hwan Lee Kyuhong<br>- 발행 : 2022	
4	1%	[카피킬러 DB] www.ncbi.nlm.nih.gov<br>- 파일명 : Protective Effects of Nintedanib against Polyhexamethylene ...<br>- 발행 : www.ncbi.nlm.nih.gov	
5	1%	[카피킬러 DB] www.ncbi.nlm.nih.gov<br>- 파일명 : Discovery and Biological Evaluations of Halogenated 2,4-Diphenyl ...<br>- 발행 : www.ncbi.nlm.nih.gov	
6	1%	[카피킬러 DB] www.copykiller.com<br>- 발행 : 인하대학교,	
7	1%	[카피킬러 DB] Copykiller<br>- 파일명 : Metabotropic Effect of Probiotic Supplementation and High-Intensity Interval Training in Menopause-Induced Metabolic Syndrome in Rats<br>- 저자 : Zeinab Bayat Arsalan Damirchi Meysam Hasannejad-Bibalan Parvin Babaei<br>- 발행 : 2023	
8	1%	[카피킬러 DB] Copykiller<br>- 파일명 : Effects of stabilizer magnesium nirate on CMIT/MIT-induced respiratory toxicity<br>- 저자 : Song Mi-Kyung Baek Yong-Wook Kim Dong Im Yoon Sung-Hoon Lee Kyuhong<br>- 발행 : 2023	
9	1%	[카피킬러 DB] Copykiller<br>- 파일명 : Hepatocytes infected with hepatitis C virus change immunological features in the liver microenvironment<br>- 저자 : Soo-Jeung Park, Young S. Hahn Hepatocytes infected with hepatitis C virus change immunological features in the liver microenvironment	
10	1%	[카피킬러 DB] Copykiller<br>- 파일명 : Immunodysregulatory potentials of polyethylene or polytetrafluorethylene microplastics to mice subacutely exposed via intragastric intubation<br>- 저자 : Jo JiHun Acharya Manju K C Pramod Bahadur Maharjan Anju Lee DaEun Gautam Ravi Kwon Jung-Taek Kim KilSoo Kim ChangYul Heo Yong Kim HyoungAh<br>- 발행 : 2023	
11	1%	[카피킬러 DB] www.mdpi.com<br>- 파일명 : Sustainability ǀ Free Full-Text ǀ High-Efficiency Microplastic Sampling Device Improved Using CFD Analysis	
12	1%	[카피킬러 DB] www.ncbi.nlm.nih.gov<br>- 파일명 : Evaluation of Animal Models by Comparison with Human Late ...<br>- 저자 : Bu-Yeo Kim, Hye-Sun Lim, Yoonju Kim, Yu Jin Kim, Imhoi Koo, Soo-Jin Jeong<br>- 발행 : 2018	

Copyright ⓒ MUHAYU All Rights reserved.

13	1%	[카피킬러 DB] Copykiller<br>- 파일명 : Ac2-26, an Annexin A1 Peptide, Attenuates Ischemia-Reperfusion-Induced Acute Lung Injury<br>- 저자 : Wen-I Liao, Shu-Yu Wu, Guofeng Wu, H Y Pao, shih-en Tang, Kun-lun Huang, Seong Jun Chu<br>- 발행 : 2017
14	1%	[카피킬러 DB] www.mdpi.com<br>- 파일명 : Ac2-26, an Annexin A1 Peptide, Attenuates Ischemia ... - MDPI
15	1%	[카피킬러 DB] www.nature.com<br>- 파일명 : Similarity of therapeutic networks induced by a multi-component ...<br>- 저자 : Bu-Yeo Kim, Hye-Sun Lim, Yu Jin Kim, Eunjin Sohn, Yun Hee Kim, Imhoi Koo, Soo-Jin Jeong<br>- 발행 : 2020/02/14
16	1%	[카피킬러 DB] Copykiller<br>- 파일명 : Design of drug crystals-based long-acting injectable systems to modulate pharmacokinetic profile and local inflammation reaction = 결정형 약물 기반 장기 지속형 주사제 설계를 통한 약물동태와 국소 염증 조절 연구<br>- 저자 : Myoung Jin Ho Design of drug crystals-based long-acting injectable systems to modulate pharmacokinetic profile and local inflammation reaction = 결정형 약물 기반 장기 지속형 주사제 설계를 통한 약물동태와 국소 염증 조절 연구
17	1%	[카피킬러 DB] Copykiller<br>- 파일명 : Monosodium iodoacetate-induced monoarthritis develops differently in knee versus ankle joint in rats<br>- 저자 : Ängeby Möller, Kristina; Klein, Stephanie; Seeliger, Frank; Finn, Anja; Stenfors, Carina; Svensson, Camilla I.<br>- 발행 : 2019-08
18	1%	[카피킬러 DB] Copykiller<br>- 파일명 : Investigating the role of radiation-induced GDF15 in human endothelial cells = 혈관세포에서 방사선 조사로 유도되는 GDF15의 역할 규명<br>- 저자 : Hyejin Park Investigating the role of radiation-induced GDF15 in human endothelial cells = 혈관세포에서 방사선 조사로 유도되는 GDF15의 역할 규명
19	1%	[카피킬러 DB] Copykiller<br>- 파일명 : Recommended levels of calcium and non-phytate phosphorus for yellow-feathered broilers (finisher phase)<br>- 저자 : Yibing Wang, Weiwei Wang, Sheng Zhang, Shouqun Jiang Recommended levels of calcium and non-phytate phosphorus for yellow-feathered broilers (finisher phase)
20	1%	[카피킬러 DB] Copykiller<br>- 파일명 : Functional importance of O-GlcNAc modification in antidepressant-like effect and autophagy<br>- 저자 : Yoonjeong Cho Functional importance of O-GlcNAc modification in antidepressant-like effect and autophagy
21	1%	[카피킬러 DB] Copykiller<br>- 파일명 : The Wnt/β-catenin signaling pathway plays a role in drug-induced liver injury by regulating cytochrome P450 2E1 expression<br>- 저자 : Shin Yoo-Sub Hwang Da-Bin Won Dong-Hoon Kim Shin-Young Kim Changuk Park Jun Won Jeon Young Yun Jun-Won<br>- 발행 : 2023
22	1%	[카피킬러 DB] www.copykiller.com<br>- 발행 : 인하대학교,
23	1%	[카피킬러 DB] Copykiller<br>- 파일명 : Cathepsin B maturation plays a critical role in leptin-induced hepatic cancer cell growth through activation of NLRP3 infl ammasomes<br>- 발행 : 2023
24	1%	[카피킬러 DB] www.copykiller.com<br>- 발행 : 인하대학교,
25	1%	[카피킬러 DB] Copykiller<br>- 파일명 : Associations of Particulate Matter Exposures With Brain Gray Matter Thickness and White Matter Hyperintensities: Effect Modification by Low-Grade Chronic Inflammation<br>- 저자 : Cho Jaelim Jang Heeseon Noh Young Lee Seung-Koo Koh Sang-Baek Kim Sun-Young Kim Changsoo<br>- 발행 : 2023
26	1%	[카피킬러 DB] Copykiller<br>- 파일명 : Naloxone ameliorates imiquimod-induced psoriatic skin inflammation by the inhibition of IL-6<br>- 저자 : IL-6Rα interaction = Imiquimod로 유발된 건선 모델에서 IL-6

Copyright ⓒ MUHAYU All Rights reserved.

27	1%	[카피킬러 DB] Copykiller<br>- 파일명 : Study on mechanisms of cell-cycle arrest and apoptosis induced by Toxoplasma gondii dense granule protein 16 (GRA16) in cancer cells [전자자료] = 암세포에서 톡소포자충 과립 단백질 16 (GRA16)이 유도하는 세포주기 억제와 세포사멸에 대한 기전 규명 연구<br>- 저자 : Seung-Hwan Seo Study on mechanisms of cell-cycle arrest and apoptosis induced by Toxoplasma gondii dense granule protein 16 (GRA16) in cancer cells [전자자료] = 암세포에서 톡소포자충 과립 단백질 16 (GRA16)이 유도하는 세포주기 억제와 세포사멸에 대한 기전 규명 연구
28	1%	[카피킬러 DB] Copykiller<br>- 파일명 : Lactobacillus rhamnosus JY02 Ameliorates Sarcopenia by Anti-Atrophic Effects in a Dexamethasone-Induced Cellular and Murine Model<br>- 저자 : Lee Juyeon Kang Minkyoung Yoo Jiseon Lee Sujeong Kang Minji Yun Bohyun Kim Jong Nam Moon Hyoungsun Chung Yihyung Oh Sangnam<br>- 발행 : 2023
29	1%	[카피킬러 DB] Copykiller<br>- 파일명 : 활성 및 억제 약물-표적 상호작용 예측을 위한 기계학습 모델 개발과 검증 = Development and validation of a machine learning model for predicting activatory and inhibitory drug-target interactions<br>- 저자 : 李圓融 활성 및 억제 약물-표적 상호작용 예측을 위한 기계학습 모델 개발과 검증 = Development and validation of a machine learning model for predicting activatory and inhibitory drug-target interactions
30	1%	[카피킬러 DB] Copykiller<br>- 파일명 : Potential of mycobacterium tuberculosis chorismate mutase (Rv1885c) as a novel TLR4-mediated adjuvant for dendritic cell-based cancer immunotherapy [전자자료] = 수지상세포 기반 항암면역요법을 위한 새로운 TLR4 매개 보조제로서의 결핵균 유래 코리스메이트...<br>- 저자 : 정혜인 Potential of mycobacterium tuberculosis chorismate mutase (Rv1885c) as a novel TLR4-mediated adjuvant for dendritic cell-based cancer immunotherapy [전자자료] = 수지상세포 기반 항암면역요법을 위한 새로운 TLR4 매개 보조제로서의 결핵균 유래 코리스메이트 뮤테이즈 Rv1885c의 가능성 연구

Copyright ⓒ MUHAYU All Rights reserved.

검사 문서	비교 문서
문장표절률: **42%**<br>Pulmonary toxicity assessment of polypropylene, polystyrene, and polyethylene microplastic fragments in mice. Isaac Kwabena Danso1,2, Jong-Hwan Woo1,3, Seung Hoon Baek1,2, Kilsoo Kim4, 5, Kyuhong Lee1,2,* 1 Inhalation Toxicology Center for Airborne Risk Factor, Korea Institute of Toxicology, 30 Baehak1-gil, Jeongeup, Jeollabuk-do, 56212, Republic of Korea	[www.ncbi.nlm.nih.gov] Polypropylene nanoplastic exposure leads to lung inflammation through p38-mediated NF-κB pathway due to mitochondrial damage - PMC<br>Hwan Woo Hyeon Jin Seo 1 Inhalation Toxicology Center for Airborne Risk Factor, Korea Institute of Toxicology, 30 Baehak1-gil, Jeongeup, Jeollabuk-do 56212 Republic of Korea Find articles by Hyeon Jin Seo Jun-Young Lee1Inhalation Toxicology Center for Airborne Risk Factor, Korea Institute of Toxicology, 30 Baehak1-gil, Jeongeup, Jeollabuk-do 56212 Republic of Korea Find articles by Jun-Young ...... by Bumseok Kim Kyuhong Lee1Inhalation Toxicology Center for Airborne Risk Factor, Korea Institute of Toxicology, 30 Baehak1-gil, Jeongeup, Jeollabuk-do 56212 Republic of Korea 3Department of Human and Environmental ...... and License information PMC Disclaimer 1 Inhalation Toxicology Center for Airborne Risk Factor, Korea Institute<br><br>[Copykiller] Effects of stabilizer magnesium nirate on CMIT/MIT-induced respiratory toxicity<br>저자 : Song Mi-Kyung Baek Yong-Wook Kim Dong Im Yoon Sung-Hoon Lee Kyuhong<br>발행 : 2023<br>to this work. Kyuhong Lee ********@********* 1 Inhalation Toxicology Center for Airborne Risk Factor, Korea Institute of Toxicology, 30 Baekhak1-Gil, Jeongeup, Jeollabuk-Do
문장표절률: **51%**<br>2 Department of Humanand Environmental Toxicology, Korea National University of Science & Technology, Daejeon, 34113, Republic of Korea 3 Biosafety Research Institute and Laboratory of Pathology, College of Veterinary Medicine, Jeonbuk National University, Iksan-si, Jeollabuk do, Republic of Korea	[www.ncbi.nlm.nih.gov] Oral administration of Jinan Red Ginseng and licorice extract ...<br>저자 : Daram Yang, Hyuneui Jeong, Seung-Mi Hwang, Jong-Won Kim, Hee-Won Moon, Ye-Eun Lee, Hyo-Bin Oh, Chung-berm Park, Bumseok Kim<br>발행 : January 2022<br>Bumseok Kima,* Daram Yang aBiosafety Research Institute and Laboratory of Pathology, College of Veterinary Medicine, Jeonbuk National University, Iksan-si, Jeollabuk do, Republic of Korea Find articles by Daram Yang Hyuneui Jeong aBiosafety Research Institute and Laboratory of Pathology, College of Veterinary Medicine, Jeonbuk National University, Iksan-si, Jeollabuk do, Republic of Korea Find articles by Hyuneui Jeong ...... Hwang Jong-Won Kim aBiosafety Research Institute and Laboratory of Pathology, College of Veterinary Medicine, Jeonbuk National University, Iksan-si, Jeollabuk do, Republic of Korea Find articles by Jong-Won ...... Kim Hee-Won Moon aBiosafety Research Institute and Laboratory of Pathology, College of Veterinary Medicine, Jeonbuk National University, Iksan-si, Jeollabuk do, Republic of Korea Find articles by Hee-Won<br><br>[www.ncbi.nlm.nih.gov] Polypropylene nanoplastic exposure leads to lung inflammation through p38-mediated NF-κB pathway due to mitochondrial damage - PMC<br>Kisoo Jeon Bumseok Kim 2Biosafety Research Institute and Laboratory of Pathology, College of Veterinary Medicine, Jeonbuk National University, Iksan-Si, Jeollabuk do Republic of Korea Find articles by Bumseok Kim ...... 56212 Republic of Korea 2Biosafety Research Institute and Laboratory of Pathology, College of Veterinary Medicine, Jeonbuk National University, Iksan-Si, Jeollabuk do Republic of Korea 3Department of Humanand Environmental Toxicology
문장표절률: **67%**<br>4 Preclinical Research Center, Daegu-Gyeongbuk Medical Innovation Foundation, Daegu 41061, Republic of Korea 5 College of Veterinary Medicine, Kyungpook National University, 80 Dahakro, Buk-gu, Daegu 41566, Republic of Korea * Corresponding author.	[www.ncbi.nlm.nih.gov] Discovery and Biological Evaluations of Halogenated 2,4-Diphenyl ...<br>발행 : www.ncbi.nlm.nih.gov<br>2 New Drug Development Center Daegu-Gyeongbuk Medical Innovation Foundation Daegu 41061 Republic of Korea. 3 College of Pharmacy, Graduate ...... of Korea. 4 Laboratory Animal Center Daegu-Gyeongbuk Medical Innovation Foundation Daegu 41061 Republic of Korea 5 College of Veterinary Medicine Kyungpook National University Daegu 41566 Republic of Korea<br><br>[Copykiller] Immunodysregulatory potentials of polyethylene or polytetrafluorethylene microplastics to mice subacutely exposed via intragastric intubation<br>저자 : Jo JiHun Acharya Manju K C Pramod Bahadur Maharjan Anju Lee DaEun Gautam Ravi Kwon Jung-Taek Kim KilSoo Kim ChangYul Heo Yong Kim HyoungAh<br>발행 : 2023<br>Research, Incheon, Republic of Korea 4 Preclinical Research Center, Daegu-Gyeongbuk Medical Innovation Foundation, Daegu, Republic of Korea 5 College of Veterinary Medicine, Kyungpook National University, Daegu, Republic of Korea 6
문장표절률: **0%**<br>E-mail addresses: khleekit@gmail.com (K. Lee) Tel: +82-63-570-8740, Fax: +82-63-570-8798 ABSTRACT Polypropylene (PP), polystyrene (PS), and polyethylene (PE) plastics are commonly used in household items such as electronic housings, food packaging, bottles, bags, toys, and roofing membranes.	

문장표절률: 0%

The presence of inhalable microplastics in indoor air has become a topic of concern as many people spent extended periods of time indoors during the COVID-19 pandemic lockdown restrictions, however, the toxic effects on the respiratory system are not properly understood.

문장표절률: 0%

We examined the toxicity of PP, PS, and PE microplastic fragments in the pulmonary system of C57BL/6 mice. For 14 days, mice were intratracheally instilled 5 mg/kg PP, PS, and PEdaily.

문장표절률: 30%

The number of inflammatory cells such as macrophages, neutrophils, and eosinophils in thebronchoalveolar lavage fluid (BALF) of PS-instilled mice was significantly higher than that in the vehicle control (VC).

[Copykiller] Design of drug crystals-based long-acting injectable systems to modulate pharmacokinetic profile and local inflammation reaction = 결정형 약물 기반 장기 지속형 주사제 설계를 통한 약물동태와 국소 염증 조절 연구

저자 : Myoung Jin Ho Design of drug crystals-based long-acting injectable systems to modulate pharmacokinetic profile and local inflammation reaction = 결정형 약물 기반 장기 지속형 주사제 설계를 통한 약물동태와 국소 염증 조절 연구

E stained tissues. The total number of inflammatory cells such as macrophages, neutrophils, and monocytes along with the inflammatory

문장표절률: 0%

The levels of inflammatory cytokines and chemokines in BALF of PS-instilled mice increased compared to the VC. However, the inflammatory responses in PP- and PE-stimulated mice were not significantly different from those in the VC group.

문장표절률: 0%

We observed elevated protein levels of toll-like receptor (TLR) 2 in the lung tissue of PP-instilled mice and TLR4 in the lung tissue of PS-instilled mice compared with those to the VC, while TLR1, TLR5, and TLR6 protein levels remained unchanged.

문장표절률: 0%

Phosphorylation of nuclear factor kappa B (NF-κB) and IκB-α increased significantly in PS-instilled mice compared with that in VC.

문장표절률: 52%

Furthermore, Nucleotide-binding oligomerization domain-like receptor family pyrin domain-containing 3 (NLRP3) inflammasome components including NLRP3, apoptosis-associated speck-like protein containing a caspase recruitment domain (ASC), and Caspase-1 in the lung tissue of PS-instilled mice increased compared with that in the VC, but not in PP- and PE-instilled mice.

[Copykiller] Hepatocytes infected with hepatitis C virus change immunological features in the liver microenvironment

저자 : Soo-Jeung Park, Young S. Hahn Hepatocytes infected with hepatitis C virus change immunological features in the liver microenvironment

activation of inflammasomes such as nucleotide-binding oligomerization domain-like receptor family pyrin domain containing 3 (NLRP3), apoptosis-associated speck-like protein containing a caspase recruitment domain (ASC), caspase-1, and release of

[www.ncbi.nlm.nih.gov] Protective Effects of Nintedanib against Polyhexamethylene ...

발행 : www.ncbi.nlm.nih.gov

is composed of NLRP3, an apoptosis-associated speck-like protein containing a caspase recruitment domain (ASC), and cysteine protease caspase-1 [22

문장표절률: 0%

These results suggest that PS microplastic fragmentstimulation induces pulmonary inflammation due to NF-κB and NLRP3 inflammasome activation by the TLR4 pathway.

문장표절률: 0%

Keywords Microplastic; Fragment; TLR4; NF-κB; NLRP3 inflammasome. 1. Introduction Microplastics have become ubiquitous in the environment owing to the increasing production and consumption of plastic products, coupled with inadequate disposal and slow biodegradation [1-3].

문장표절률: 0%

Microplastics exist primarily as purposefully manufactured micro-sized products, or secondary microplastics formed from the disintegration of plastic debris exposed to ultraviolet radiation, mechanical stress, and biological actions in the environment [4].

Copyright ⓒ MUHAYU All Rights reserved.

문장표절률: 0%

As a result, the occurrence of microplastics in the environment is diverse, consisting of a wide range of polymer types such as polypropylene (PP), polystyrene (PS), and polyethylene (PE), with different sizes and shapes including beads, fragments, and f ibres [2, 5].

문장표절률: 0%

Microplastic pollution has been previously discussed as a marine environmental iss ue. However, in recent times, its occurrence in both indoor and outdoor air enviro nments has been confirmed, with reports suggesting that inhalation is a more domi nant route of exposure [6-9].

문장표절률: 0%

Although research on airborne microplastic exposure has primarily focused on out door environments, a limited number of studies have revealed that indoor micropla stic concentrations are notably elevated.

문장표절률: 0%

These elevated indoor microplastic levels may exert adverse effects on human healt h [10-12]. Furthermore, the COVID-19 pandemic and associated lockdown regul ations have led to prolonged indoor periods for individuals, underscoring the signif icance of this study.

문장표절률: 0%

Despite the potential risks involved, our understanding of the pulmonary toxicity o f inhaled microplastics in humans remains limited.

문장표절률: 0%

In recent studies, the fragment and fibre shapes in atmospheric microplastics were observed to be the most predominant, and it has also been reported that humans a re exposed to an average of 55,000 particles annually via the inhalation route [13-15].

문장표절률: 0%

In addition, microplastics of 12 polymer types including PP, PS, and PE were detect ed in the lung tissue of humans, with most of them being fragments (43%) and fibr e (49%) [16].

문장표절률: 0%

Microplastic particles have also been observed in the sputum of patients with vario us respiratory diseases [17]. Previous studies have reported that some occupational workers with long-term exposure to microplastics developed pulmonary diseases i ncluding lung cancer and asthma [2, 3, 18-21]. Although some studies have high l ighted the potential health risks associated with

문장표절률: 0%

the inhalation of microplastics, our understanding of the toxicity mechanisms of dif ferent types of microplastics within the respiratory system remains limited.

문장표절률: 26%

Toll-like receptors (TLRs) are pattern recognition receptors (PRRs) that detect bot h exogenous pathogen-associated molecular patterns (PAMPs) and endogenous da nger-associated molecular patterns (DAMPs), thereby triggering host defense resp onses and inflammations [22-24].

[patents.google.com] Conjugated TLR7 and/or TLR8 and TLR2 polycationic a gonists

발행 : 2013-02-22, 2014-08-27

subject. BACKGROUND OF THE INVENTION Toll-like receptors (TLRs) are p attern recognition receptors (PRRs) expressed by diverse cell types

문장표절률: 0%

TLRs can activate multiple intracellular signals, including the nuclear factor κB (NF-κB) pathway, which plays a major role in regulating inflammatory responses [25].

문장표절률: 0%

Recent research has indicated that NF-κB serves as an integral component of the i nitiation signal required for stimulation of the NLRP3 inflammasome, which subse quently triggers the activation of Caspase-1 and consequent release of interleukin (IL)-1β [26, 27].

Copyright ⓒ MUHAYU All Rights reserved.

문장표절률: **0%**

TLR-mediated inflammation is implicated in pulmonary diseases including asthma , pulmonary fibrosis, and chronic obstructive pulmonary disease (COPD) [28-32].

문장표절률: **0%**

Recent research has reported that air pollution agents such as particulate matter, a ctivated TLR2- and TLR4-mediated lung inflammations [33-35], however, the m echanism of pathogenesis for lung inflammation due to microplastic exposure is stil l unclear.

문장표절률: **0%**

This study investigated inflammatory responses including inflammatory cytokine a nd chemokine levels, cellular changes, and histopathological analysis of bronchoal veolar lavage fluid (BALF) and lung tissue of PP-, PS-, and PE-instilled mice.

문장표절률: **0%**

Additionally, we examined the mechanism of toxicity of TLR-mediated lung infla mmation in the lung tissues of microplastic-instilled mice.

문장표절률: **0%**

2. Material and methods 2.1 PP, PS, and PE microplastic fragments PP beads (Prop ylTex® 50, Micro Powders Inc., NewYork, NY, USA) and PE beads (5 mm) were purchased to prepare PP and PE microplastic fragments of size approximately 〈 20 µm.

문장표절률: **0%**

PP and PE beads were frozen at −78℃ and subsequently ground into a powder usi ng a blade-shaped homogenizer,

문장표절률: **0%**

a process lasting approximately 4 h. PP and PE beads were frozen at −78℃ and su bsequently homogenized to a powder using a blade-shaped device; this process las ted approximately 4 h.

문장표절률: **0%**

The resulting powder was passed through a 50-µm mesh and washed five times w ith ethanol. The samples were then dried at 50℃ for 48 h.

문장표절률: **0%**

To produce PP and PE microplastics with particlesizes of approximately 〈20 µm, microplastics were dispersed in ethanol and subjected to high-pressure homogeniz ation (four passes at 600 bar).

문장표절률: **0%**

Subsequently, the resulting particles were passed through 15-µm mesh filters, subj ected to five ethanol washes, and then dried for 48 h at 50℃.

문장표절률: **0%**

PS microplastic fragments were supplied by the Korea Testing and Research Institute. To prepare microplastic fragments for experimentation, PP, PS, and PE m icroplastics were dispersed in a solution consisting of 1% DMSO in saline.

문장표절률: **0%**

The resulting solution was sonicated in a water bath for 30 min. Field-EmissionSc anning Electron Microscopy (FE-SEM) (S-4800, Hitachi, Japan) analysis was per formed to determine the shape and size of the three microplastic fragments.

문장표절률: **0%**

Zeta potentials (ELSZ-2000, Otsuka, Japan) were measured in triplicate to deter mine their respective surface charges.

Copyright ⓒ MUHAYU All Rights reserved.

문장표절률: **20%**

2.2 Animals and experimental design Male C57BL/6 mice of seven weeks old were procured from Orient Bio Inc. (Seongnam, Korea) and housed in a controlled envi ronment at constant temperature and relative humidity of 22 ± 3 ℃ and 50 ± 20 % respectively, and a 12 h light/dark cycle.

[Copykiller] Comparative toxicity of polyhexamethylene guanidine phosphate i n three strains of rats

저자 : Woo Jong-Hwan Kim Ki Cheon Kim Hyeon-Young Kim In-Hyeon Kim Sung-Hwan Lee Kyuhong
발행 : 2022

Wistar rats were pur- chased from Orient Bio Inc. (Seongnam, Korea) and housed in a temperature-controlled environment 22 ±

문장표절률: **0%**

Throughout the experiment, mice were provided with standard experimental roden t pellets (PMI Nutrition International, Richmond, IN, USA) and UV-sterilized and filtered tap water ad libitum.

문장표절률: **71%**

Experimental procedures were carried out with approval from the Institutional Ani mal Care and Use Committee at the Korea Institute of Toxicology (IACUC #2108 -0023).

[www.ncbi.nlm.nih.gov] Polypropylene nanoplastic exposure leads to lung infla mmation through p38-mediated NF-κB pathway due to mitochondrial damag e - PMC

experimental procedures were approved by the Institutional Animal Care and Use Committee at the Korea Institute of Toxicology (IACUC #2108-0003). The mice i n the

[www.copykiller.com] [2023-04-07] Role of NDRG3 in regulating CDK1 acti vation and tumor growth in ovarian cancer.docx

발행 : 인하대학교, 2023.04.

model All animal experiments were approval from the Institutional Animal Care a nd Use Committee (IACUC) at Inha University in

문장표절률: **0%**

In the PP, PS, and PE experimental groups, mice were subjected to intratracheal ins tillation of 5 mg/kg of PP, PS, and PE suspended in a 50 µl saline solution over th e 2 weeks, employing an automatic instillator [36].

문장표절률: **0%**

Similarly, mice in the vehicle control (VC) group were also intratracheally instilled with saline. Mice of all groups were sacrificed on day 15.

문장표절률: **0%**

2.3 Bronchoalveolar lavage fluid (BALF) analysis At 24 h after the administration of the three microplastics (PP, PS, and PE), the mice were anesthetized using isoflu rane and euthanized by exsanguination.

문장표절률: **0%**

The left lung was ligated and the trachea cannulated. Subsequently, the right lung was lavaged three times, with 0.7 mL of phosphate-buffered saline (PBS).

문장표절률: **43%**

Total cells of the collected BALF werecounted with the help of a NucleoCounter (NC-250; ChemoMetec, Gydevang, Denmark). BALF cell smears were prepared fo r differential cell counts using Cytospin (Thermo Fisher Scientific) and stained wit h Diff-Quik solution (Dade Diagnostics, Aguada, Puerto, USA). A total of 200 cell s werecounted per slide.

[www.ncbi.nlm.nih.gov] Polypropylene nanoplastic exposure leads to lung infla mmation through p38-mediated NF-κB pathway due to mitochondrial damag e - PMC

types, BAL cells were prepared using Cytospin (Thermo Fisher Scientific) and stain ed with Diff-Quik solution (Dade Diagnostics, Aguada, Puerto, USA). A total of 2 00 cells werecounted on each slide. Measurement of

[Copykiller] Effects of stabilizer magnesium nirate on CMIT/MIT-induced resp iratory toxicity

저자 : Song Mi-Kyung Baek Yong-Wook Kim Dong Im Yoon Sung-Hoon Lee Kyuhong
발행 : 2023

Cytospin (Thermo Fisher Scientific Inc and stained with Diff-Quik solution (Dade Diagnostics, Aguada, Puerto Rico). The different cell types

Copyright ⓒ MUHAYU All Rights reserved.

문장표절률: **36%**

2.4 Histopathological analysis The left lung tissue of mice were fixed in 10% neutral-buffered formalin. The tissuespecimens were dehydrated and embedded in paraffin.

[www.copykiller.com] 윤석영 2020년 8월 박사학위논문초본.docx

발행 : 인하대학교, 2020.05.

lung parenchyma, the left lungs of mice were fixed in 10% neutral buffered formalin and embedded in paraffin. Sections

[www.copykiller.com] 윤석영 박사1.docx

발행 : 인하대학교, 2019.11.

lung parenchyma, the left lungs of mice were fixed in 10% neutral buffered formalin and embedded in paraffin. Sections

문장표절률: **89%**

Subsequently, sections (4- μm-thick) were stained with hematoxylin and eosin (H&E). All fields per section from each animal were analyzed using a Leica DM2500 microscope (Leica Instruments, Wetzlar, Germany) at 200x and 400x magnifications.

[Copykiller] Comparative toxicity of polyhexamethylene guanidine phosphate in three strains of rats

저자 : Woo Jong-Hwan Kim Ki Cheon Kim Hyeon-Young Kim In-Hyeon Kim Sung-Hwan Lee Kyuhong

발행 : 2022

s tri- chrome (MT)-stain. All fields per section from each animal were analyzed using a Leica DM2500 microscope (Leica instruments, Wetzlar, Germany) at 200x magnification. The degree of lung

[www.ncbi.nlm.nih.gov] Polypropylene nanoplastic exposure leads to lung inflammation through p38-mediated NF-κB pathway due to mitochondrial damage - PMC

E). All samples were analysed using a Leica DM2500 microscope (Leica Instruments, Wetzlar, Germany) at 200x and 400x magnifications. The degree of lung injury

문장표절률: **61%**

2.5 Inflammatory cytokine and chemokine levels in BALF The levels of IL-1β, IL-6, monocyte chemoattractant protein-1 (MCP-1), macrophage inflammatory protein (MIP)-1α, MIP-2, and C-X-C motif chemokine ligand 1 (CXCL1/KC) in BALF were measured using commercial ELISA kits (R&D System) according to the manufacturer's instructions.

[Copykiller] 활성화된 대식세포주에서 Sabinene의 항염증 효능 평가 = Anti-inflammatory effect of Sabinene in LPS-induced RAW264.7cell

저자 : 서승이

2(COX-2),TNF- α,IL-1β, IL-6,Monocyte chemoattractant protein-1 (MCP-1/CCL2)과 같은 염증성 사이토카인의

[www.ncbi.nlm.nih.gov] Polypropylene nanoplastic exposure leads to lung inflammation through p38-mediated NF-κB pathway due to mitochondrial damage - PMC

factor-α (TNF-α), interleukin IL 1β, IL-6, monocyte chemoattractant protein-1 (MCP-1), and C-X-C motif chemokine ligand 1 (CXCL1/KC) were significantly increased in the

문장표절률: **0%**

2.6 Preparation of protein extract and western blot analysis Lung tissues were homogenized using RIPA buffer (Thermo Fisher Scientific) supplemented with a protease and phosphatase inhibitor cocktail, following the manufacturer's instructions.

문장표절률: **0%**

Protein concentrations were quantified using the Bradford reagent (Bio-Rad). The samples were subsequently loaded and separated using sodium dodecyl sulfate-polyacrylamide gel electrophoresis at 90 V for 120 min.

문장표절률: **0%**

Following electrophoresis, the samples were transferred by the wet method unto polyvinylidene difluoride membranes (Merck Millipore) at a current of 250 mA for 60 min.

문장표절률: **0%**

After blocking non-specific sites with 5% non-fat dry milk in 0.1% Tween 20 in Tris-buffered saline (TBS-T) for 1 h, the membrane

문장표절률: **0%**

was incubated overnight at 4 ℃ with TLR1 (Abeomics, San Diego, USA), TLR2 (Abcam, Cambridge, UK), TLR4 (Invitrogen, Massachusetts, USA), TLR5 (Abcam, Cambridge, UK), TLR6 (Boster Bio, Pleasanton, USA), NF-κB (Cell Signaling, Massachusetts, USA), p-NF-κB (Cell Signaling, Massachusetts, USA), IκB (Cell Signaling, Massachusetts, USA), p-IκB (Cell Signaling, Massachusetts, USA), NLRP3 (AdipoGen Life Sciences, Inc.

Copyright ⓒ MUHAYU All Rights reserved.

문장표절률: 35%	[www.copykiller.com] 손미권 박사학위 논문.hwp
Liestal, Switzerland), ASC (AdipoGen Life Sciences, Inc. Liestal, Switzerland), Caspase-1 (AdipoGen Life Sciences, Inc. Liestal, Switzerland), and β-actin (Santa Cruz Biotechnology, Dallas, TX, USA).	발행 : 인하대학교, 2015.07.<br>Proteintech, Chicago, IL, USA), GAPDH, and β-actin (Santa Cruz Biotechnology, Dallas, TX, USA). Secondary antibodies were purchased from

문장표절률: 18%	[www.ncbi.nlm.nih.gov] Polypropylene nanoplastic exposure leads to lung inflammation through p38-mediated NF-κB pathway due to mitochondrial damage - PMC
Horseradish peroxidase-linked anti-rabbit IgG (Cell Signaling, Massachusetts, USA) and anti-mouse IgG (Cell Signaling, Massachusetts, USA) were used to detect antibody binding and with the help of iBright CL 1000 imaging system (Thermo Fisher Scientific), bands were visualized after treatment with the ECL reagent (Thermo Fisher Scientific).	imaging system (Thermo Fisher Scientific) after treatment with the ECL reagent (Thermo Fisher Scientific). Theresults of the densitometric analysis

문장표절률: 94%	[www.ncbi.nlm.nih.gov] Polypropylene nanoplastic exposure leads to lung inflammation through p38-mediated NF-κB pathway due to mitochondrial damage - PMC
The results of the densitometric analysis were expressed as the relative ratio of the target protein to the reference protein. The relative ratio of the target protein to the control was arbitrarily denoted as 1.	The results of the densitometric analysis were expressed as the relative ratio of the target protein to the reference protein. The relative ratio of the target protein to the control was arbitrarily denoted
	[www.mdpi.com] Ac2-26, an Annexin A1 Peptide, Attenuates Ischemia ... - MDPI
	USA). The data were presented as the relative ratio of the target protein to the reference protein. The relative ratio of the target protein of the control group was

문장표절률: 100%	[Copykiller] Comparative toxicity of polyhexamethylene guanidine phosphate in three strains of rats
2.7 Statistical analysis All statistical analyses were performed using GraphPad InStat v. 3.0 (GraphPad Software, Inc., La Jolla, CA, USA).	저자 : Woo Jong-Hwan Kim Ki Cheon Kim Hyeon-Young Kim In-Hyeon Kim Sung-Hwan Lee Kyuhong<br>발행 : 2022<br>200 x magnifi cationStatistical analysis Statistical analyses were performed using GraphPad InStat v. 3.0 (GraphPad Software, Inc., La Jolla, CA, USA). Data are presented as mean
	[Copykiller] [논문]Bacillus subtilis가 함유된 목질계 인공토양의 물리·화학적 특성이 참싸리 생육에 미치는 영향
	저자 : 목재공학 = Journal of the Korean wood science and technology v.47 no.4 , 2019년, pp.393 - 407 Kim, Ji-Su (Division of Environmental Forest Science, Major of Environmental Materials Science, Institute of Agriculture & Life Science, Gyeongsang National University) ; Jung, Ji young (Division of Environmental Forest Science, Major of Environmental Materials Science, Institute of Agriculture & Life Science, Gyeongsang National University) ; Ha, Si Young (Division of Environmental Forest Science, Major of Environmental Materials Science, Institute of Agriculture & Life Science, Gyeongsang National University) ; Yang, Jae-Kyung (Division of Environmental Forest Science, Major of Environmental Materials Science, Institute of Agriculture & Life Science, Gyeongsang National University)<br>발행 : 2019<br>2.7 Statistical analysis All statistical analyses were performed using IBM SPSS statistics v25 (IBM

문장표절률: 64%	[Copykiller] Lactobacillus rhamnosus JY02 Ameliorates Sarcopenia by Anti-Atrophic Effects in a Dexamethasone-Induced Cellular and Murine Model
Statistical comparisons between more than two groups were performed using one-way analysis of variance (ANOVA) followed by Dunnett's multiple comparison test, and statistical comparisons between two groups were conducted using Student's t-test.	저자 : Lee Juyeon Kang Minkyoung Yoo Jiseon Lee Sujeong Kang Minji Yun Bohyun Kim Jong Nam Moon Hyoungsun Chung Yihyung Oh Sangnam<br>발행 : 2023<br>Statistical analyses were performed using one-way analysis of variance (ANOVA) followed by Dunnett's test and Tukey's multiple
	[Copykiller] 활성 및 억제 약물-표적 상호작용 예측을 위한 기계학습 모델 개발과 검증 = Development and validation of a machine learning model for predicting activatory and inhibitory drug-target interactions
	저자 : 李圓融 활성 및 억제 약물-표적 상호작용 예측을 위한 기계학습 모델 개발과 검증 = Development and validation of a machine learning model for predicting activatory and inhibitory drug-target interactions<br>deviation. Statistical significance was determined using one-way analysis of variance (ANOVA) followed by Dunnett's multiple comparison post-hoc test. Statistical significance

Copyright ⓒ MUHAYU All Rights reserved.

문장표절률: **89%**

Data arepresented as the mean ± SD. A value of p 〈 0.05 was considered to indicatestatistically significant results.

[www.ncbi.nlm.nih.gov] Polypropylene nanoplastic exposure leads to lung inflammation through p38-mediated NF-κB pathway due to mitochondrial damage - PMC

Data arepresented as the arithmetic mean ± SD. A value of p 〈 0.05 was considered to indicatestatistically significant results.

[Copykiller] [논문]NDRG2 Expression Decreases Tumor-Induced Osteoclast Differentiation by Down-regulating ICAM1 in Breast Cancer Cells

저자 : Biomolecules & therapeutics v.24 no.1 , 2016년, pp.9 - 18 Kim, Bomi (Department of Biological Science and the Research Center for Women's Disease, Sookmyung Women's University) ; Nam, Sorim (Department of Biological Science and the Research Center for Women's Disease, Sookmyung Women's University) ; Lim, Ji Hyun (Department of Biological Science and the Research Center for Women's Disease, Sookmyung Women's University) ; Lim, Jong-Seok (Department of Biological Science and the Research Center for Women's Disease, Sookmyung Women's University)
발행 : 2016

statistical analysis. Values are represented as the mean ± SD. A value of p 〈 0.05 was considered significant. RESULTS Osteoclast differentiation is

문장표절률: **0%**

3. Results 3.1 Characterization of PP, PS, and PE microplastic fragments To ascertain the detailed size and shape of threekind of microplastic fragments, each sample was bath sonicationed for 0.5 h and then studied by FE-SEM image (Fig.

문장표절률: **0%**

1). The microplastic particles generally appeared as irregular fragmentshapes. The results revealed that the microplastic fragments had a diameter of 6.40 ± 1.48 μm for PP (Fig. 1a). Additionally, the averagediameters of PS and PE

출처표시 문장 문장표절률: **0%**

microplastics were 17.53 ± 2.11 μm and 21.27 ± 6.07 μm, respectively (Fig. 1b-c). The zeta potential of PP, PS, and PE fragments was -8.28 ± 1.37, -38.93 ± 4.49, and -5.71 ± 1.10 mV, respectively (Table 1). 3.2 Inflammatory response in PP, PS, and PE-stimulated mice

문장표절률: **0%**

We examined the inflammatory response to PP, PS, and PE microplastic fragmentstimulation. Our results showed that total cells, macrophages, neutrophils, and eosinophils were significantly increased in the PS stimulation mice compared to those in the VC (Fig.

문장표절률: **0%**

2a). The percentage of macrophages in the BALF of PS-instilled mice decreased significantly to 79.75% compared to that in VC.

문장표절률: **0%**

However, the neutrophil percentage (11.00%) and eosinophil percentage (9.25%) were significantly higher than those in VC (Fig. 2b).

문장표절률: **0%**

The inflammatory cellular changes in the BALF of PP- and PE-instilled mice were not significantly different from those in the VC (Fig.

문장표절률: **0%**

2). Histopathological analysis of the lung tissues of PP-, PS-, and PE-instilled mice showed inflammatory cell infiltration.

문장표절률: **23%**

In addition, the PS-stimulated mice showed increased macrophage infiltration (Fig. 3). Furthermore, the levels of inflammatory cytokines such as IL-1β and IL-6 in the BALF of PS-instilled mice were higher than those in the VC, but not in PP- and PE-instilled mice (Fig.

[kaken.nii.ac.jp] 急性期重症患者擬似モデルにおける高血糖惹起性腸内細菌動態と対応策

resulted in the up-regulation of inflammatory cytokines such as IL-1β and IL-6 in mesenteric lymph nodes, and of

문장표절률: 0%

4a-b). Our results showed that the levels of inflammatory chemokines including MCP-1, MIP-1α, MIP-2, and KC increased in the 5 mg/kg PS-instilled mice co mpared to those in the VC; however, the inflammatory chemokine levels in PP- a nd PE-instilled mice remained significantly unchanged (Fig. 4c-f).

문장표절률: 0%

3.3 Microplastic fragmentstimulation induces TLRs activation TLRs are a group of proteins involved in the early stages of the host defense against invading pathogens , which is an important factor in the regulation of inflammatory response [22-25].

문장표절률: 0%

We investigated the protein levels of TLRs in the lung tissue of microplastic-instille d mice. Our results showed that PS-instilled mice had significantly increased expre ssion levels of TLR4 as compared to the VC group.

문장표절률: 0%

However, the levels of TLR1, 2, 5, and 6 in PS-instilled mice remained unchanged compared to those in the VC group (Fig. 5). Interestingly, PP-instilled mice showe d a significant increase in the

문장표절률: 0%

expression levels of TLR2 as compared to the VC group. The expression levels of TLR1, 4, 5, and 6 did not significantly increase in PP-instilled mice (Fig.

문장표절률: 0%

5). In PE-instilled mice, the protein levels of all TLRs were not significantly differe nt from those in VC (Fig. 5).

문장표절률: 0%

3.4 PS microplastic fragments stimulation activates NLRP3 Inflammasome through NF-κB signaling pathway Our results showed that the p-IκB-α protein levels i n the lung tissue of PS-instilled mice weresignificantly increased compared to those in VC.

문장표절률: 48%

Additionally, the p-NF-κB protein levels in the lung tissue of PS-stimulated mice increased compared to those in the VC (Fig.

[www.ncbi.nlm.nih.gov] Polypropylene nanoplastic exposure leads to lung infla mmation through p38-mediated NF-κB pathway due to mitochondrial damag e - PMC

4a-d). The p-NF-κB protein levels in the lung tissue of 5 mg/kg PP-instilled

문장표절률: 0%

6). However, the protein levels of IκB-α and NF-κB phosphorylation in the lun g tissues of PP- and PE-instilled mice remained unchanged (Fig.

문장표절률: 0%

6). Furthermore, we observed the protein levels of NLRP3 inflammasome compone nts such as NLRP3, ASC, and Caspase-1. Our results showed that NLRP3, ASC, and Caspase-1 expression weresignificantly increased in the lung tissue of PS-stim ulated mice compared to that in the VC, while PP- and PE-instilled mice did not s how significant changes (Fig. 7).

문장표절률: 0%

4. Discussion We investigated the molecular mechanism of the pulmonary toxicity response to PP, PS, and PE microplastic fragmentstimulation in mice.

문장표절률: 0%

Our results showed the inflammatory response including inflammatory cells, cytok ines, and chemokines in BALF of PS intratracheal instillation mice increased compa red to the VC.

문장표절률: 0%

Histopathological analysis of the lung tissue of PS-instilled mice revealed lung inju ry such as inflammatory infiltration in the perivascular/peribronchial region.

Copyright ⓒ MUHAYU All Rights reserved.

문장표절률: 0%

The PS fragments stimulation significantly increased the protein levels of TLR4 in t he lung tissue with respect to the VC, but not protein levels of TLR1, 2, 5, and 6.

문장표절률: 0%

The protein levels of IκB-α and NF-κB phosphorylation in the lung tissue of PS -treated mice were significantly increased compared to those in VC.

문장표절률: 0%

PS stimulation led to a significant increase in NLRP3 inflammasome components i ncluding NLRP3, ASC, and Caspase-1. These results suggest that PS microplastic fragments may contribute to NF-κB and NLRP3-mediated inflammation via the TLR4 signaling pathway in the respiratory system.

문장표절률: 0%

The detrimental effects of airborne microplastics on the pulmonary system have be en rarely reported. Although in vitro and in vivo studies have demonstrated threats , the variations in the characteristics of environmental microplastics require wider t oxicity investigations for a better understanding.

문장표절률: 0%

Previous studies have explored the effects of microplastic exposure on different pol ymers in the pulmonary system. Specifically, 6.25 mg/kg of PS microplastic intratr acheal instillation in mice has been observed to induce pulmonary inflammation [3 7].

문장표절률: 0%

In another study, the administration of PS microplastic via intranasal instillation da ily at a dose of 40 mg/kg for 21 days resulted in a significant increase in the levels of inflammatory cytokines in the lungs of mice [38].

문장표절률: 0%

PE microplastics have also been reported to exert inflammatory effects on mouse l ungs at concentrations ranging between 500 to 2,000 mg/kg after 28 days of expos ure via oral administration.

문장표절률: 0%

In this study, the no-observed-adverse-effect level (NOAEL) was estimated to be less than 1,000 mg/kg in male mice and 〈 500 mg/kg in female mice [39].

문장표절률: 0%

An evaluation of the toxicity of polypropylene fragments through oral administrati on in mice showed that the NOAEL for PP microplastics was greater than 2,000 m g/kg [40].

문장표절률: 0%

Nonetheless, it is worth noting that intratracheal instillation of PP microplastic in mice induces inflammation at a dose of 2.5 mg/kg, as demonstrated in a previous s tudy [41].

문장표절률: 0%

This discrepancy in microplastic doses among toxicity assessments highlights the v ariations in our understanding of their effects.

문장표절률: 0%

Furthermore, the environmental concentrations of microplastics tend to vary depen ding on the catchment area. A recent study in Shanghai estimated that daily human exposure to inhalable indoor aerosols is approximately 704 ± 254 microplastic ite ms with approximately 526 ± 203 microplastic items deposited in the pulmonary airway [42].

Copyright ⓒ MUHAYU All Rights reserved.

문장표절률: **0%**

In other parts, microplastic concentrations range between 230 ± 94 and 358 ± 13 2 items/m3, mostly as fragments and fibres [43].

문장표절률: **0%**

Generally, adult humans inhale approximately 6.5–8.97 μg/kg microplastics daily . However, this rate can be significantly higher in infants, ranging from 3 to 50 tim es the adult levels [44].

문장표절률: **0%**

Therefore, we observed the pulmonary toxic effects of 5 mg/kg (daily concentratio n) in PP-, PS-, and PE-instilled mice.

문장표절률: **0%**

Previous studies have revealed various physiological dysfunctions caused by micro plastic exposure in vivo and in vitro [38,41,45,46].

문장표절률: **0%**

These effects depend on factors such as size and shape [3]. Using FE-SEM imagin g, we confirmed the shapes and sizes of PP, PS, and PE microplastic fragments. PP, PS,

문장표절률: **0%**

and PE particles were observed to have relatively irregular morphologies with aver age sizes of about 6.40 ± 1.48 μm, 17.53 ± 2.11 μm, and 21.27 ± 6.07 μm respe ctively (Fig.

문장표절률: **0%**

1). Studies have reported that microplastic exposure induces toxicity in various bio logical systems in a size-dependent manner [5,47].

문장표절률: **0%**

However, in this study, PS-instilled mice showed significantly higher toxicity resp onses, including an increase in cellular recruitment and inflammatory cytokine and chemokine levels than PP- and PE-instilled mice (Fig.

문장표절률: **0%**

2 and 4), although PP microplastic fragments were the smallest. We hypothesized t hat a combination of other additional properties such as surface charges might acc ount for these responses.

문장표절률: **0%**

PS particles were known to exhibit relatively higher levels of hardness and brittlene ss as compared to PP and PE, also recent studies have reported that PS is more likel y to induce cellular system dysfunctions accompanied by the infiltration of leukocy tes via physical abrasion [38, 39].

문장표절률: **0%**

Moreover, PS was synthesized from the free radical polymerization of styrene, a de rivative of benzene, with benzoyl peroxide as the initiator, and these chemicals wer e known to be a toxic effect, also which might lead to physiological dysfunction du e to the chemical releases [40, 41].

문장표절률: **0%**

Owing to their elevated surface-to-volume ratio, the surface charges of microplast ics have been reported to play a crucial role in influencing their functions and inter actions within biological systems, potentially resulting in adverse effects [48].

문장표절률: **0%**

Zeta potential measurements provide information about particle charges and dispe rsion stability, with absolute values above 30 mV indicating low aggregation that l eads to good homogenization following exposure [49,50].

Copyright ⓒ MUHAYU All Rights reserved.

문장표절률: 0%

Although the role of surface charge in microplastic toxicity has been sparsely reported, inhalation of negatively charged PS microplastic (zeta potential of − 35.98 ± 0.26 mV) reportedly induced an influx of leukocytes and inflammatory cytokine expression in BALF and lung tissues of mice [51].

문장표절률: 0%

In accordance with the findings of Shao et al., polymeric particles that share similar charges, irrespective of whether negative or positive, tend to exhibit increased cytotoxicity and enhanced affinity for cells as their charge values increase [52].

출처표시 문장 문장표절률: 0%

Similarly, the mean zeta potentials recorded for PP, PS, and PE fragments in this study were −8.28 ± 1.37, −38.93 ± 4.49, and −5.71 ± 1.10 mV, respectively (Table 1), following a toxicity trend of PE (−5.71 ± 1.10 mV) 〈 PP (−8.28 ± 1.37 mV) 〈 PS (−38.93 ± 4.49 mV).

문장표절률: 0%

The interplay of these properties may contribute to the inflammatory response for microplastic exposure. Hence, further studies are imperative to gain a comprehensive understanding

문장표절률: 0%

of the precise roles and mechanisms through which these physicochemical characteristics influence both short-term and long-term exposure effects.

문장표절률: 0%

Immune cells play an essential role in homeostasis maintenance in the lung by recognizing and eliminating inhaled foreign substances; however, excessive infiltration of inflammatory cells may cause lung injury [53–55].

문장표절률: 38%

Our results showed that PS stimulation significantly increased the number of inflammatory cells including macrophages, neutrophils, and eosinophils, in the BALF of mice (Fig.

[www.ncbi.nlm.nih.gov] Polypropylene nanoplastic exposure leads to lung inflammation through p38-mediated NF-κB pathway due to mitochondrial damage - PMC

Our results showed that the number of inflammatory cells including macrophages, neutrophils, and lymphocytes in the BALF of

문장표절률: 0%

2). In addition, the levels of inflammatory chemokines, including MCP-1, MIP-1α, MIP-2, and KC in the BALF of PS-instilled mice significantly increased compared to those in the VC (Fig.

문장표절률: 0%

4c–f). Previous studies have reported that airborne particles such as particulate matter increased the number of macrophages, neutrophils, and eosinophils in the BALF of mice and the levels of inflammatory chemokines, including MCP-1 were increased compared to those in the VC [56].

문장표절률: 0%

MCP-1 is a key chemokine involved in the migration and infiltration of monocytes/macrophages and also plays a role in the recruitment of eosinophils to acute and chronic inflammatory sites [57,58].

문장표절률: 0%

Recent studies reported that diesel exhaust particlestimulation increased the number of neutrophils and the levels of inflammatory cytokines and chemokines such as IL-6, MCP-1, MIP-2, and KC in the lung tissue of mice [59].

문장표절률: 0%

The chemokines MIP-2 and KC are linked to the influx of neutrophils in the rodent lung [60], both of which have been implicated in the inflammatory process [61,62].

Copyright ⓒ MUHAYU All Rights reserved.

문장표절률: 0%

These results suggest that PS stimulation causes cellular recruitment and inflammatory cytokine and chemokine release, which might lead to pulmonary inflammation in the respiratory system.

문장표절률: 0%

TLRs are essential components of the innate immune system against invading pathogens through their recognition of molecular patterns and subsequent initiation of the inflammatory response [33-35].

문장표절률: 0%

Recent studies have reported that most air pollution agents, such as particulate matter, induce inflammations through TLR2- and TLR4-mediated signaling, which is detected by the endogenous DAMP ligands released by tissue injury [63-65].

문장표절률: 0%

Recent studies have demonstrated differences in the functions of TLRs. TLR1, TLR2, and TLR6 require heterodimer formation such as TLR1/TLR2 and TLR2/TLR6 for the activation of inflammatory responses including IL-1β secretion, whereas TLR4

문장표절률: 0%

activates the inflammatory responses as a homodimer [66-68]. TLRs initiate signaling pathways that result in the nuclear translocation of NF-κB and NLRP3 inflammasome activation, which play an essential role in the pathogenesis of lung inflammation via cytokine release and mediators [63].

문장표절률: 0%

We examined the protein levels of TLRs in the lung tissues of PP, PS, and PE microplastic fragment-stimulated mice. The TLR2 level in the lung tissue of PP-stimulated mice significantly increased compared with that in the VC, but the levels of TLR1, TLR4, TLR5, and TLR6 did not increase (Fig.

문장표절률: 0%

5). PP-instilled mice showed no change in protein levels of p-IκB-α, p-NF-κB, and NLRP3 inflammasome components compared with those in the VC (Fig.

문장표절률: 0%

6 and 7). These results show that PP stimulation increases the TLR2 level; however, the absence of TLR1 and TLR6 might result in no heterodimer formation.

문장표절률: 0%

On the other hand, PS stimulation significantly increased the protein level of TLR4 (Fig. 5). The protein levels of p-IκB-alpha and p-NF-κB in the lung tissues of PS-instilled mice significantly increased compared to the VC (Fig.

문장표절률: 0%

6). In addition, a significant increase in the protein levels of NLRP3 inflammasome components including NLRP3, ASC, and Caspase-1 of lung tissue in PS-stimulated mice as compared to the VC (Fig.

문장표절률: 0%

7). These results showed that PS microplastic fragmentstimulation may induce pulmonary inflammation linked to NLRP3 and NF-κB through the TLR4 signaling pathway.

문장표절률: 80%

Author contributions IKD: Methodology, Conceptualization, Formal analysis, Investigation, Writing-original draft, Writing-review and editing.

[www.ncbi.nlm.nih.gov] Polypropylene nanoplastic exposure leads to lung inflammation through p38-mediated NF-κB pathway due to mitochondrial damage - PMC

Vehicle control Author contributions JHW: Methodology, Conceptualization, Formal analysis, Investigation, Writing-original draft, Writing-review and editing. SHJ: Methodology, Formal analysis, Investigation

Copyright © MUHAYU All Rights reserved.

문장표절률: **0%**

JHW: Methodology, Formal analysis, Investigation, Writing-review and editing. SHB: Methodology, Formal analysis, Investigation, Writing-review and editing.

문장표절률: **79%**

KK: Methodology, Formal analysis, Investigation. LK: Conceptualization, Writing-review and editing, Supervision, Funding acquisition, Project administration. All authors read and approved the final manuscript.

[www.ncbi.nlm.nih.gov] Polypropylene nanoplastic exposure leads to lung inflammation through p38-mediated NF-κB pathway due to mitochondrial damage - PMC

Writing-review and editing, Supervision. LK: Conceptualization, Writing-review and editing, Supervision, Funding acquisition, Project administration. All authors read and approved the final manuscript. Funding This work was supported

문장표절률: **97%**

Funding This work was supported by Korea Environment Industry & Technology Institute (KEITI) through the Measurement and Risk assessment Program for Management of Microplastics Project, funded by Korea Ministry of Environment (MOE) (grant number HE-2305).

[www.ncbi.nlm.nih.gov] Polypropylene nanoplastic exposure leads to lung inflammation through p38-mediated NF-κB pathway due to mitochondrial damage - PMC

Funding This work was supported by Korea Environment Industry & Technology Institute (KEITI) through the Measurement and Risk assessment Program for Management of Microplastics Project, funded by Korea Ministry of Environment (MOE) (grant number HE

[www.copykiller.com] JCR_Full paper_V3_kor.docx

발행 : 인하대학교, 2023.03.

구조적 건전성을 확인해야 한다. ACKNOWLEDGMENTS This work was supported by Korea Environment Industry & Technology Institute funded by Korea Ministry of

문장표절률: **76%**

Data availability All datasets generated during the current study are available from the corresponding author on request.

[iatriki-sfakianakis.blogspot.com] Transplantation

발행 : blogspot

generated during and/or analyzed during the current study are available from the corresponding author on reasonable request. Clinical trial notation

[www.copykiller.com] 2. Manuscript track change version-표절검사.docx

발행 : 인하대학교, 2022.02.

materials used and/or analyzed during the current study are available from the corresponding author on reasonable request.

문장표절률: **58%**

Declarations Conflict of interest All authors confrm that there is no confict of interest. Ethics approval All experimental procedures were approved by the Institutional Animal Care and Use Committee of the Korea Institute of Toxicology (IACUC #2108-0023).

[www.ncbi.nlm.nih.gov] Protective Effects of Nintedanib against Polyhexamethylene ...

발행 : www.ncbi.nlm.nih.gov

The experimental procedures were approved by the Institutional Animal Care and Use Committee of the Korea Institute of Toxicology (IACUC #1708-0306). 4.2. Test

[www.ncbi.nlm.nih.gov] Evaluation of Animal Models by Comparison with Human Late ...

저자 : Bu-Yeo Kim, Hye-Sun Lim, Yoonju Kim, Yu Jin Kim, Imhoi Koo, Soo-Jin Jeong
발행 : 2018

approved the final manuscript. Notes Ethics Approval All experimental procedures were approved by the Institutional Animal Care and Use Committee of the Korea Institute of Oriental Medicine and were performed

참고문헌

References 1. Barnes, D.K.A., Galgani, F., Thompson, R.C., Barlaz, M. (2009) Accumulation and fragmentation of plastic debrisinglobal environments. Philos. Trans. R. Soc. B. Biol. Sci., 364, 1985-1998. https://doi.org/10.1098%2Frstb.2008.0205 2. Kannan, K., Vimalkumar, K. (2021) A Review of HumanExposure to Microplastics and Insights Into Microplastics as Obesogens. Front. Endocrinol (Lausanne)., 12, 724989. https://doi.org/10.3389/fendo.2021.724989 3. Prata, J.C. (2018) Airborne microplastics: Consequences to human health? Environ. Pollut., 234, 115-126. https://doi.org/10.1016/j.envpol.2017.11.043 4. Andrady, A.L. (2017) The plastic in microplastics: A review. Mar. Pollut. Bull., 119, 12-22. https://doi.org/10.1016/j.marpolbul.2017.01.082 5. Schwarzer, M., Brehm, J., Vollmer, M., Jasinski, J., Xu, C., Zainuddin, S., Frohlich, T., Schott, M., Greiner, A., Scheibel, T., Laforsch, C. (2022) Shape, size, and polymer dependent effects of microplastics on Daphnia magna. J. Hazard. Mater., 425, 128136. https://doi.org/10.1016/j.jhazmat.2021.128136

6. Cai, L., Wang, J., Peng, J., Tan, Z., Zhan, Z., Tan, X., Chen, Q. (2017) Charact eristic of microplastics in the atmospheric fallout from Dongguan city, China: preli minary research and first evidence. Environ, Sci. Pollut. Res., 24, 24928–24935. htt ps://doi.org/10.1007/s11356-017-0116-x 7. Wright, S.L., Ulke, J., Font, A., Cha n, K.L.A., Kelly, F.J. (2020) Atmospheric microplastic deposition in an urban envir onment and an evaluation of transport. Environ. Int., 136, 105411. https://doi.org /10.1016/j.envint.2019.105411 8. Dris, R., Gasperi, J., Saad, M., Mirande, C., Tass in, B. (2016) Synthetic fibers in atmospheric fallout: A source of microplastics in th e environment? Mar. Pollut. Bull., 104, 290–293. https://doi.org/10.1016/j.marpo lbul.2016.01.006 9. Chen, Q., Gao, J., Yu, H., Su, H., Yang, Y., Cao, Y., Zhang, Q., Ren, Y., Hollert, H., Shi, H., Chen, C., Liu, H. (2022) An emerging role of mic roplastics in the etiology of lung ground glass nodules. Environ. Sci. Eur., 34, 25. h ttps://doi.org/10.1186/s12302-022-00605-3

10. Liao, Z., Ji, X., Ma, Y., Lv, B., Huang , W., Zhu, X., Fang, M., Wang, Q., Wa ng, X., Dahlgren, R., Shang, X. (2021) Airborne microplastics in indoor and outdo or environments of a coastal city in Eastern China. J. Hazard. Mater., 417, 126007 . https://doi.org/10.1016/j.jhazmat.2021.126007 11. Dris, R., Gasperi, J., Mirande , C., Mandin, C., Guerrouache, M., Langlois, V., Tassin, B. (2017) A first overview of textile fibers, including microplastics, in indoor and outdoor environments. Envi ron. Pollut., 221, 453–458. https://doi.org/10.1016/j.envpol.2016.12.013 12. Zhai , X., Zheng, H., Xu, Y., Zhao, R., Wang, W., Guo, H. (2023) Characterization an d quantification of microplastics in indoor environments. Heliyon., 9, e15901. http s://doi.org/10.1016/j.heliyon.2023.e15901 13. Cox, K.D., Covernton, G.A., Davie s, H.L., Dower, J.F., Juanes, F., Dudas, S.E. (2019) Human Consumption of Micro plastics. Environ. Sci. Technol., 53, 7068–7074. https://doi.org/10.1021/acs.est.9b 01517 14. Allen, S., Allen, D., Phoenix, V.R., Roux, G.L., Jimenez, P.D., Simonnea u, A., Binet, S., Galop, D. (2019) Atmospheric transport and deposition of micropl astics in a remote mountain catchment. Nat. Geosci., 12, 339–344. https://doi.org /10.1038/s41561-019-0335-5

15. Kernchen, S., Loder, M.G.J., Fischer, F., Fischer, D., Moses, S.R., Georgi, C., Nolscher, A.C., Held, A., Laforsch, C. (2022) Airborne microplastic concentrations and deposition across the Weser River catchment. Sci. Total. Envir on., 818, 151812. https://doi.org/10.1016/j.scitotenv.2021.151812 16. Jenner, L.C. , Rotchell, J.M., Bennett, R.T., Cowen, M., Tentzeris, V., Sadofsky, L.R. (2022) D etection of microplastics in human lung tissue using μFTIR spectroscopy. Sci. Tot al. Environ., 831, 154907. https://doi.org/10.1016/j.scitotenv.2022.154907 17. Hu ang, S., Huang, X., Bi, R., Guo, Q., Yu, X., Zeng, Q., Huang, Z., Liu, T., Wu, H., Chen, Y., Xu, J., Wu, Y., Guo, P. (2022) Detection and analysis of microplastics in humansputum. Environ. Sci. Technol., 56, 2476–2486. https://doi.org/10.1021/ac s.est.1c03859 18. Facciola, A., Visalli, G., Ciarello, M.P., Pietro, A.D. (2021) Newl y emerging airborne pollutants: Current knowledge of health impact of micro and nanoplastics. Int. J. Environ. Res. Public. Health., 18, 2997. https://doi.org/10.339 0%2Fijerph18062997

19. Wright, S.L., Kelly, F.J. (2017) Plastic and human health: a micro issue? Enviro n. Sci. Technol., 51, 6634–6647. https://doi.org/10.1021/acs.est.7b00423 20. Hou rs, M., Fevotte, J., Lafont, S., Bergeret, A. (2007) Cancer mortality in a synthetic s pinning plant in Besançon, France. Occup. Environ. Med., 64, 575–581. https://d oi.org/10.1136%2Foem.2006.028282 21. Turcotte, S.E., Chee, A., Walsh, R., Gran t, F.C., Liss, G.M., Boag, A., Forkert, L., Munt, P.W., Lougheed, M.D. (2013) Flo ck worker's lung disease: natural history of cases and exposed workers in Kingston, Ontario. Chest., 143, 1642–1648. https://doi.org/10.1378/chest.12-092 0 22. Kovach, M.A., Standiford, T.J. (2011) Toll like receptors in diseases of the lu ng. Int. Immunopharmacol., 11, 1399–1406. https://doi.org/10.1016%2Fj.intimp. 2011.05.013 23. Medzhitov, R. (2001) Toll-like receptors and innate immunity. N at. Rev. Immunol., 1, 135–145. https://doi.org/10.1038/35100529 24. Jiang, D., Li ang, J., Li, Y., Noble, P.W. (2006) The role of Toll-like receptors in non-infectiou s lung injury. Cell. Res., 16, 693–701. https://doi.org/10.1038/sj.cr.7310085

25. Ben, D.F., Yu, X.Y., Ji, G.Y., Zheng, D.Y., Lv, K.Y., Ma, B., Xia, Z.F. (2012) TLR4 mediates lung injury and inflammation in intestinal ischemia-reperfusion. J. Surg. Res. 174, 326–333. https://doi.org/10.1016/j.jss.2010.12.005 26. Tao, X., Li , J., He, J., Jiang, Y., Liu, C., Cao, W., Wu, H. (2023) Pinellia ternata (Thunb.) Br eit. attenuates the allergic airway inflammation of cold asthma via inhibiting the ac tivation of TLR4-medicated NF-kB and NLRP3 signaling pathway. J. Ethnophar macol., 315, 116720. https://doi.org/10.1016/j.jep.2023.116720 27. Redondo-Cas tro, E., Faust, D., Fox, S., Baldwin, A.G., Osborne, S., Haley, M.J., Karran, E., Nu thall, H., Atkinson, P.J., Dawson, L.A., Routledge, C., Allan, S.M., Freeman, S., B rownlees, J., Brough, D. (2018) Development of a characterised tool kit for the int errogation of NLRP3 inflammasome-dependent responses. Sci. Rep., 8, 5667. http s://doi.org/10.1038/s41598-018-24029-3 28. Lim, J.O., Kim, W.I., Pak, S.W., Le e, S.J., Park, S.H., Shin, I.S., Kim, J.C. (2023) Toll-like receptor 4 is a key regulator of asthma exacerbation caused by aluminum oxide nanoparticles via regulation of NF-κB phosphorylation. J. Hazard. Mater., 448, 130884. https://doi.org/10.101 6/j.jhazmat.2023.130884

Copyright ⓒ MUHAYU All Rights reserved.

29. Bolourani, S., Brenner, M., Wang, P. (2021) The interplay of DAMPs, TLR4, and proinflammatory cytokines in pulmonary fibrosis. J. Mol. Med (Berl)., 99, 1373−1384. https://doi.org/10.1007/s00109-021-02113-y 30. Pace, E., Ferraro, M., Siena, L., Melis, M., Montalbano, A.M., Johnson, M., Bonsignore, M.R., Bonsignore, G., Gjomarkaj, M. (2008) Cigarettesmoke increases Toll-like receptor 4 and modifies lipopolysaccharide-mediated responses in airway epithelial cells. Immunology., 124, 401−411. https://doi.org/10.1111%2Fj.1365-2567.2007.02788.x 31. Sidletskaya, K., Vitkina, T., Denisenko, Y. (2020) The Role of Toll-Like Receptors 2 and 4 in the Pathogenesis of Chronic Obstructive Pulmonary Disease. Int. J. Chron. Obstruct. Pulmon. Dis., 15, 1481−1493. https://doi.org/10.2147%2FCOPD.S249131 32. Zaffaroni, L., Peri, F. (2018) Recent advances on Toll-like receptor 4 modulation: New therapeutic perspectives. Future. Med. Chem., 10, 461−476. https://doi.org/10.4155/fmc-2017-0172 33. Becker, S., Dailey, L., Soukup, J.M., Silbajoris, R., Devlin, R.B. (2005) TLR-2 is involved in airway epithelial cell response to air pollution particles. Toxicol. Appl. Pharmacol., 203, 45−52. https://doi.org/10.1016/j.taap.2004.07.007

34. He, M., Ichinose, T., Yoshida, Y., Arashidani, K., Yoshida, S., Takano, H., Sun, G., Shibamoto, T. (2017) Urban PM2.5 exacerbates allergic inflammation in the murine lung via a TLR2/TLR4/MyD88-signaling pathway. Sci. Rep., 7, 11027. https://doi.org/10.1038/s41598-017-11471-y 35. Shoenfelt, J., Mitkus, R.J., Zeisler, R., Spatz, R.O., Powell, J., Fenton, M.J., Squibb, K.A., Medvedev, A.E. (2009) Involvement of TLR2 and TLR4 in inflammatory immune responses induced by fine and coarse ambient air particulate matter. J. Leukoc. Biol., 86, 303−312. https://doi.org/10.1189/jlb.1008587 36. Kim, J.S., Lee, B., Hwang, I.C., Yang, Y.S., Yang, M.J., Song, C.W. (2010) An automatic video instillator for intratracheal instillation in the rat. Lab. Anim., 44, 20−24. https://doi.org/10.1258/la.2009.009003 37. Li, X., Zhang, T., Lv, W., Wang, H., Chen, H., Xu, Q., Cai, H., Dai, J. (2022) Intratracheal administration of polystyrene microplastics induces pulmonary fibrosis by activating oxidative stress and Wnt/β-catenin signaling pathway in mice. Ecotoxicol. Environ. Saf., 232, 113238. https://doi.org/10.1016/j.ecoenv.2022.113238

38. Cao, J., Xu, R., Geng, Y., Xu, S., Guo, M. (2023) Exposure to polystyrene microplastics triggers lung injury via targeting toll-like receptor 2 and activation of the NF-κB signal in mice. Environ. Pollut., 320, 121068. https://doi.org/10.1016/j.envpol.2023.121068 39. Lee, S., Kang, K.K., Sung, S.E., Choi, J.H., Sung, M., Seong, K.Y., Lee, S., Yang, S.Y., Seo, M.S., Kim, K. (2022) Toxicity Study and Quantitative Evaluation of Polyethylene Microplastics in ICR Mice. Polymers (Basel), 14, 402. https://doi.org/10.3390/polym14030402 40. Lee, S., Kim, D., Kang, K.K., Sung, S.E., Choi, J.H., Sung, M., Shin, C.H., Jeon, E., Kim, D., Kim, D., Lee, S., Kim, H.K., Kim, K. (2023) Toxicity and Biodistribution of Fragmented Polypropylene Microplastics in ICR Mice. Int. J. Mol. Sci., 24, 8463. https://doi.org/10.3390/ijms24108463 41. Woo, J.W., Seo, H.J., Lee, J.Y., Lee, I., Jeon, K., Kim, B., Lee, K. (2023) Polypropylene nanoplastic exposure leads to lung inflammation through p38-mediated NF-κB pathway due to mitochondrial damage. Part. Fibre. Toxicol., 20, 2. https://doi.org/10.1186/s12989-022-00512-8

42.Geng, Y., Zhang, Z., Zhou, W., Shao, X., Li, Z., Zhou, Y. (2023) Individual exposure to microplastics through the inhalation route: comparison of microplastics in inhaled indoor aerosol and exhaled breath air. Environ. Sci. Technol. Lett., 10, 464−470. https://doi.org/10.1021/acs.estlett.3c00147 43. Zhu, X., Huang, W., Fang, M., Liao, Z., Wang, Y., Xu, L., Mu, Q., Shi, C., Lu, C., Deng, H., Dahlgren, R., Shang, X. (2021) Airborne microplastic concentrations in five megacities of northern and southeast china. Environ. Sci. Technol., 55, 12871−12881. https://doi.org/10.1021/acs.est.1c03618 44. Zhang, J., Wang, L., Kannan, K. (2019) Polyethylene Terephthalate and Polycarbonate Microplastics in Pet Food and Feces From the United States. Environ. Sci. Technol., 53, 12035−12042. https://doi.org/10.1021/acs.est.9b03912 45. Xia, T., Kovochich, M., Liong, M., Zink, J.I., Nel, A.E. (2008) Cationic polystyrene nanosphere toxicity depends on cell-specific endocytic and mitochondrial injury pathways. ACS. Nano. 2, 85−96. https://doi.org/10.1021/nn700256c

46. Chiu, H.W., Xia, T., Lee, Y.H., Chen, C.W., Tsai, J.C., Wang, Y.J. (2015) Cationic polystyrene nanospheres induce autophagic cell death through the induction of endoplasmic reticulum stress. Nanoscale. 7, 736−746. https://doi.org/10.1039/c4nr05509h 47. An, D., Na, J., Song, J., Jung, J. (2021) Size-dependent chronic toxicity of fragmented polyethylene microplastics to Daphnia magna. Chemosphere, 271, 129591. https://doi.org/10.1016/j.chemosphere.2021.129591 48. Zając, M., Kotyńska, J., Zambrowski, G., Breczko, J., Deptuła, P., Cieśluk, M., Zambrzycka, M., Święcicka, I., Bucki, R., Naumowicz, M. (2023) Exposure to polystyrene nanoparticles leads to changes in the zeta potential of bacterial cells. Sci. Rep., 13, 9552. https://doi.org/10.1038/s41598-023-36603-5 49. Canepari, S., Padella, F., Astolfi, M. L., Marconi, E., Perrino, C. (2013) Elemental concentration in atmospheric particulate matter: Estimation of nanoparticle contribution. Aerosol. Air. Qual. Res., 13, 1619−1629. https://doi.org/10.4209/aaqr.2013.03.0081

Copyright ⓒ MUHAYU All Rights reserved.

50. Saleh, Y., Antherieu, S., Dusautoir, R., Alleman, L. Y., Sotty, J., De Sousa, C., Platel, A., Perdrix, E., Riffault, V., Fronval, I., Nesslany, F., Canivet, L., Garçon, G ., & Lo-Guidice, J. M. (2019) Exposure to atmospheric ultrafine particles induces severe lung inflammatory response and tissue remodeling in mice. Int. J. Environ. Res. Public Health., 16, 1210. https://doi.org/10.3390/ijerph16071210 51. Jin, Y.J ., Kim, J.E., Roh, Y.J., Song, H.J., Seol, A., Park, J., Lim, Y., Seo, S., Hwang, D.Y. (2023) Characterisation of changes in global genes expression in the lung of ICR m ice in response to the inflammation and fibrosis induced by polystyrene nanoplastic s inhalation. Toxicol. Res., 39, 1-25. https://doi.org/10.1007/s43188-023-00188 -y 52. Shao, X.R., Wei, X.Q., Song, X., Hao, L.Y., Cai, X.X., Zhang, Z.R., Peng, Q., Lin, Y.F. (2015) Independent effect of polymeric nanoparticle zeta potential/su rface charge, on their cytotoxicity and affinity to cells. Cell. Prolif., 48, 465-474. h ttps://doi.org/10.1111/cpr.12192

53. Rosales, C. (2018) Neutrophil: A Cell with Many Roles in Inflammation or Se veral Cell Types? Front. Physiol. 9, 113. https://doi.org/10.3389/fphys.2018.0011 3 54. Hou, F., Xial, K., Tang, L., Xie, L. (2021) Diversity of Macrophages in Lung Homeostasis and Diseases. Front. Immunol. 12, 753940. https://doi.org/10.3389/f immu.2021.753940 55. Dworski, R., Simon, H.U., Hoskins, A., Yousefi, S. (2011) Eosinophil and neutrophil extracellular DNA traps in humanallergic asthmatic air ways. J. Allergy. Clin. Immunol. 127, 1260-1266. https://doi.org/10.1016%2Fj.jac i.2010.12.1103 56. Inoue, K., Takano, H., Yanagisawa, R., Sakurai, M., Ichinose, T., Sadakane, K., Yoshikawa, T. (2005) Effects of nano particles on antigen-relate d airway inflammation in mice. Respir. Res. 6, 106. https://doi.org/10.1186/1465 -9921-6-106 57. Deshmane, S.L., Kremlev, S., Amini, S., Sawaya, B.E. (2009) Mo nocyte chemoattractant protein-1 (MCP-1): an overview. J. Interferon. Cytokine. Res. 29, 313-326. https://doi.org/10.1089%2Fjir.2008.0027 58. Conti, P., Digioac chino, M. (2001) MCP-1 and RANTES are mediators of acute and chronic inflam mation. Allergy. Asthma. Proc. 22, 133-137. https://doi.org/10.2500/1088541017 78148737

59. Saber, A.T., Jacobsen, N.R., Bornholdt, J., Kjaer, S.L., Dybdahl, M., Risom, L. , Loft, S., Vogel, U., Wallin, H. (2006) Cytokine expression in mice exposed to dies el exhaust particles by inhalation. Role of tumor necrosis factor. Part. Fibre. Toxic ol. 3, 4. https://doi.org/10.1186%2F1743-8977-3-4 60. Haelens, A., Wuyts, A., P roost, P., Struyf, S., Opdenakker, G., Damme, J.V. (1996) Leukocyte migration an d activation by murine chemokines. Immunobiology. 195, 499-521. https://doi.or g/10.1016/s0171-2985(96)80019-2 61. Driscoll, K.E. (2000) TNFalpha and MIP-2: role in particle-induced inflammation and regulation by oxidative stress. Toxic ol Lett. 112-113, 177-183. https://doi.org/10.1016/s0378-4274(99)00282-9 62. Rao, K.M.K., Ma, J.Y., Meighan, T., Barger, M.W., Pack, D., Vallyathan, V. (200 5) Time course of gene expression of inflammatory mediators in rat lung after dies el exhaust particle exposure. Environ. Health. Perspect. 113, 612-617. https://doi. org/10.1289/ehp.7696 63. Danielsen, P.H., Bendtsen, K.M., Knudsen, K.B., Poulse n, S.S., Stoeger, T., Vogel, U. (2021) Nanomaterial- and shape-dependency of TL R2 and TLR4 mediated signaling following pulmonary exposure to carbonaceous nanomaterials in mice. Part. Fibre. Toxicol. 18, 40. https://doi.org/10.1186/s1298 9-021-00432-z

64. Lu, Y.C., Yeh, W.C., Ohashi, P.S. (2008) LPS/TLR4 signal transduction pathw ay. Cytokine. 42, 145-151. https://doi.org/10.1016/j.cyto.2008.01.006 65. Erridge , C. (2010) Endogenous ligands of TLR2 and TLR4: agonists or assistants? J. Leuk oc. Biol. 87, 989-999. https://doi.org/10.1189/jlb.1209775 66. Colleselli, K., Stiers chneider, A., Wiesner, C. (2023) An Update on Toll-like Receptor 2, Its Function and Dimerization in Pro- and Anti-Inflammatory Processes. Int. J. Mol. Sci., 24, 1 2464. https://doi.org/10.3390/ijms241512464 67. Ozinsky, A., Underhill, D.M., F ontenot, J.D., Hajjar, A.M., Smith, K.D., Wilson, C.B., Schroeder, L., & Aderem, A. (2000) The repertoire for pattern recognition of pathogens by the innate immun e system is defined by cooperation between toll-like receptors. Proc. Natl. Acad. S ci. U. S. A., 97, 13766-13771. https://doi.org/10.1073/pnas.250476497 68. Under hill, D.M., Ozinsky, A. (2002) Toll-like receptors: key mediators of microbe detec tion. Curr. Opin. Immunol., 14, 103-110. https://doi.org/10.1016/s0952-7915(0 1)00304-1

문장표절률: **0%**

Table 1. Surface charges of PP, PS, and PE microplastic fragments Microplastic Fra gments PP PS PE Zeta Potentials (mV) - 8.28 ± 1.37 - 38.93 ± 4.49 - 5.71 ± 1.1 0 Figure Legends Fig. 1 FE-SEM images of microplastic fragments (A) PP, (B) PS, and (C) PE. Scale bar 30 μm.

문장표절률: **0%**

Fig. 2 (A) Cellular changes in the BALF of mice stimulated with three microplastics (PP, PS, and PE). (B) Total and differential cells in BALF.

Copyright ⓒ MUHAYU All Rights reserved.

문장표절률: **72%**

Data arepresented as mean ± SD (n = 6–8 per group). #p ≤ 0.05; ##p ≤ 0.01 vs. VC. Fig. 3 (A) Representative H&E–stained section of lung tissue.

[www.ncbi.nlm.nih.gov] Polypropylene nanoplastic exposure leads to lung inflammation through p38–mediated NF–κB pathway due to mitochondrial damage – PMC

mean ± SD (n = 6 per group). #P ≤ 0.05; ##P ≤ 0.01 vs. VC Open in a separate window Fig. 3 a Representative H&E–stained section of lung tissue. b Histological scoring of inflammatory

[Copykiller] Metabotropic Effect of Probiotic Supplementation and High–Intensity Interval Training in Menopause–Induced Metabolic Syndrome in Rats

저자 : Zeinab Bayat Arsalan Damirchi Meysam Hasannejad–Bibalan Parvin Babaei
발행 : 2023

≤ 0.001 versus sham group. $P ≤ 0.05, $$P ≤ 0.01 versus OVX + Exe +

문장표절률: **0%**

Black and red arrows indicate inflammatory cell infiltration and macrophage increased. Scale bar 100 μm.

문장표절률: **20%**

Fig. 4 Inflammatory cytokines & chemokines levels, including (A) IL–1β, (B) IL–6, (C) MCP–1, (D) MIP–1α, (E) MIP–2, and (F) KC in the BALF of mice instilled with PP, PS, and PE microplastic fragments. Data arepresented as mean ± SD (n = 6–8 per group). #p ≤ 0.05; ##p ≤ 0.01 vs. VC.

[www.ncbi.nlm.nih.gov] Polypropylene nanoplastic exposure leads to lung inflammation through p38–mediated NF–κB pathway due to mitochondrial damage – PMC

mean ± SD (n = 6 per group). #P ≤ 0.05; ##P ≤ 0.01 vs. VC Open in a separate window

[Copykiller] Metabotropic Effect of Probiotic Supplementation and High–Intensity Interval Training in Menopause–Induced Metabolic Syndrome in Rats

저자 : Zeinab Bayat Arsalan Damirchi Meysam Hasannejad–Bibalan Parvin Babaei
발행 : 2023

≤ 0.001 versus sham group. $P ≤ 0.05, $$P ≤ 0.01 versus OVX + Exe +

문장표절률: **0%**

Fig. 5 (A) Representative western blotting analysis and relative density of TLRs 1, 2, 4, 5, and 6 in the lung tissue of PP–, PS–, and PE–instilled mice.

문장표절률: **0%**

Data were normalized against β–actin. Data arepresented as mean ± SD (n = 6–8 per group). ##p ≤ 0.01 vs. VC.

문장표절률: **0%**

Fig. 6 (A) Representative western blot analysis of p–IκB–α, IκB–α, p–NF–κB, and NF–κB in the lung tissue of PP–, PS–, and PE–instilled mice.

문장표절률: **58%**

(B) Relative density analysis of p–IκB–α levels. Data were normalized against IκB–α. (C) Relative density analysis of p–NF–κB levels.

[www.ncbi.nlm.nih.gov] Polypropylene nanoplastic exposure leads to lung inflammation through p38–mediated NF–κB pathway due to mitochondrial damage – PMC

were normalized against JNK. e Relative density analysis of p–IκB–α levels. Data were normalized against IκB–α. Data are means ± SD

문장표절률: **0%**

Data were normalized against NF–κB. Data arepresented as mean ± SD (n = 6–8 per group). ##p ≤ 0.01 vs. VC.

문장표절률: **0%**

Fig. 7 (A) Representative western blot analysis of NLRP3, ASC, and Caspase–1 in lung tissue of PP–, PS–, and PE–instilled mice. (B) Relative density analysis of NLRP3 levels.

Copyright ⓒ MUHAYU All Rights reserved.

문장표절률: **29%**

(C) Relative density analysis of ASC levels. (D) Relative density analysis of Caspase−1 levels. Data were normalized against β−actin. Data arepresented as mean ± SD (n = 6−8 per group). #p ≤ 0.05; ##p ≤ 0.01 vs. VC.

[www.ncbi.nlm.nih.gov] Polypropylene nanoplastic exposure leads to lung inflammation through p38−mediated NF−κB pathway due to mitochondrial damage − PMC

mean ± SD (n = 6 per group). #P ≤ 0.05; ##P ≤ 0.01 vs. VC Open in a separate window

[Copykiller] Metabotropic Effect of Probiotic Supplementation and High−Intensity Interval Training in Menopause−Induced Metabolic Syndrome in Rats

저자 : Zeinab Bayat Arsalan Damirchi Meysam Hasannejad−Bibalan Parvin Babaei
발행 : 2023

≤ 0.001 versus sham group. $P ≤ 0.05, $$P ≤ 0.01 versus OVX + Exe +

Copyright ⓒ MUHAYU All Rights reserved.
