# Supplementary figures and images for: Pulmonary toxicity assessment of polypropylene, polystyrene, and polyethylene microplastic fragments in mice
Source: Toxicol Res. 2024 Mar 8;40(2):313–23. doi: 10.1007/s43188-023-00224-x (PMC10959865; doi:10.1007/s43188-023-00224-x)

TLR1

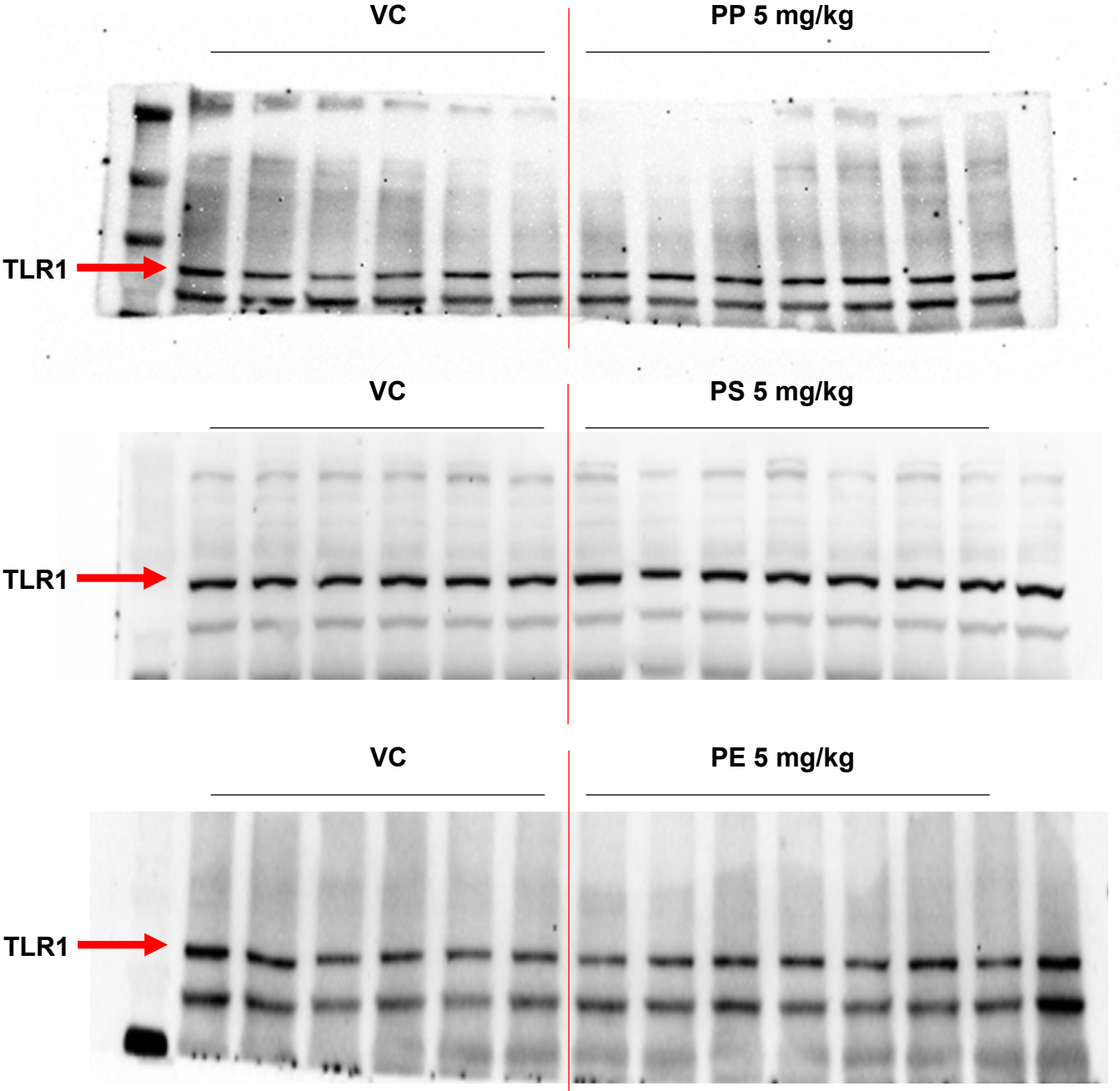

TLR2

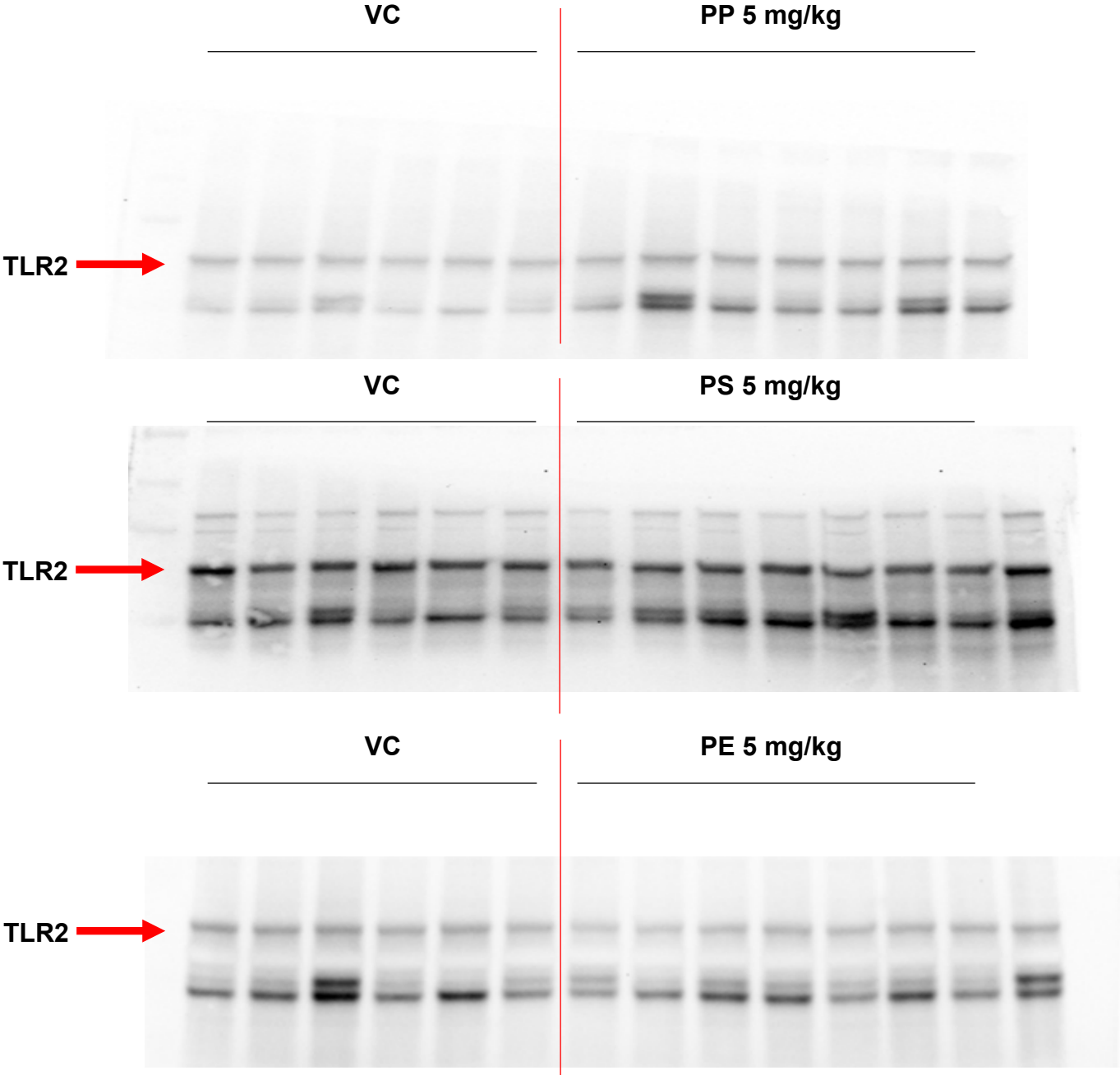

TLR4

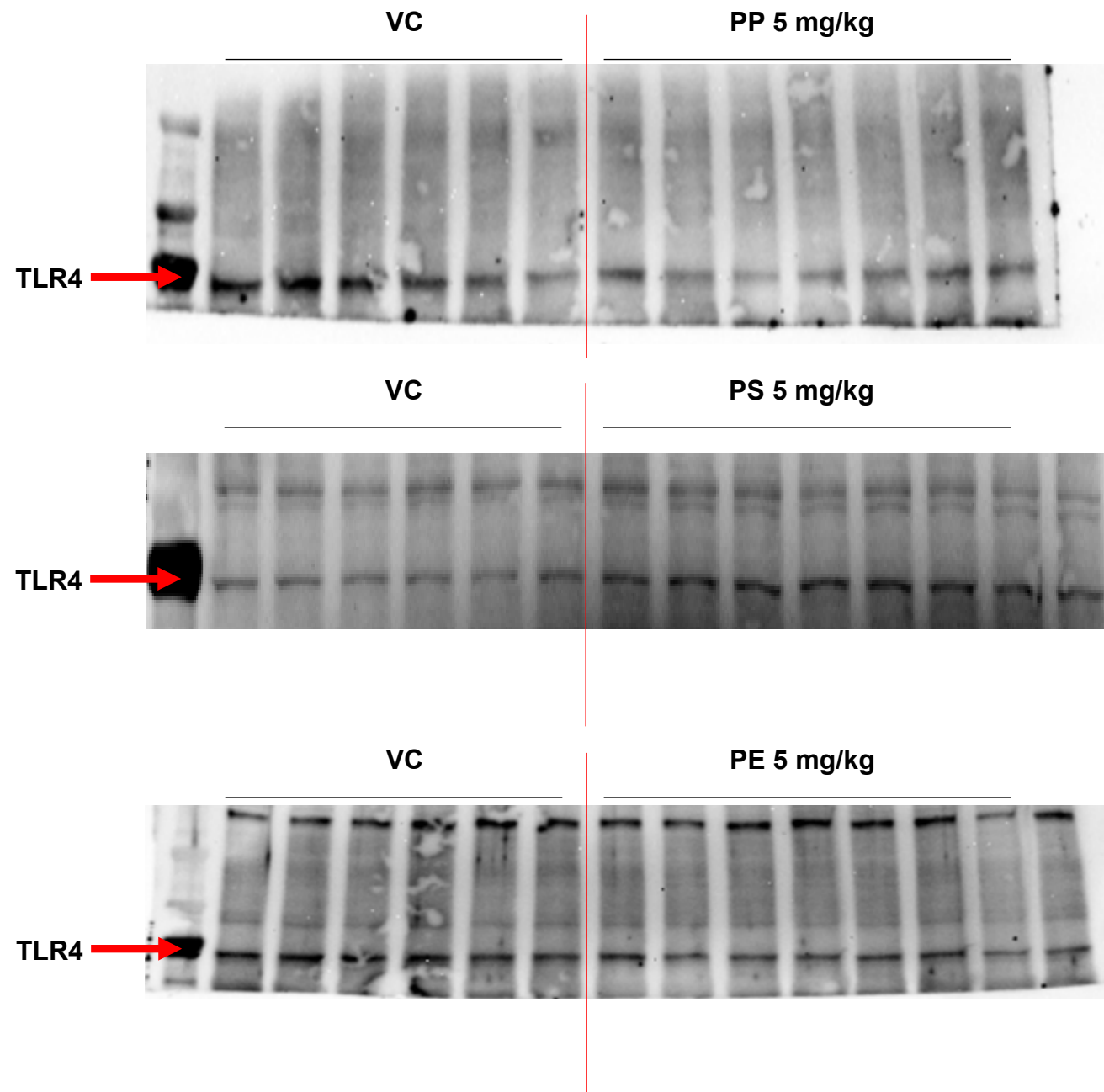

TLR5

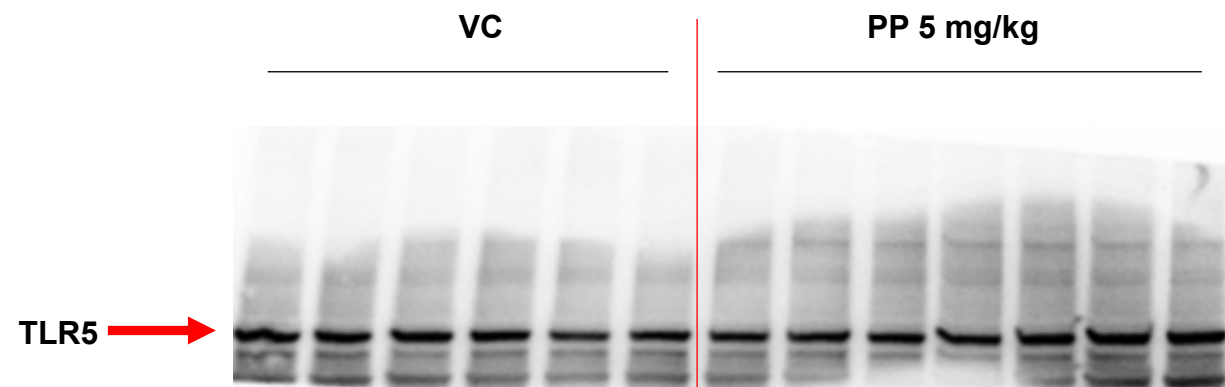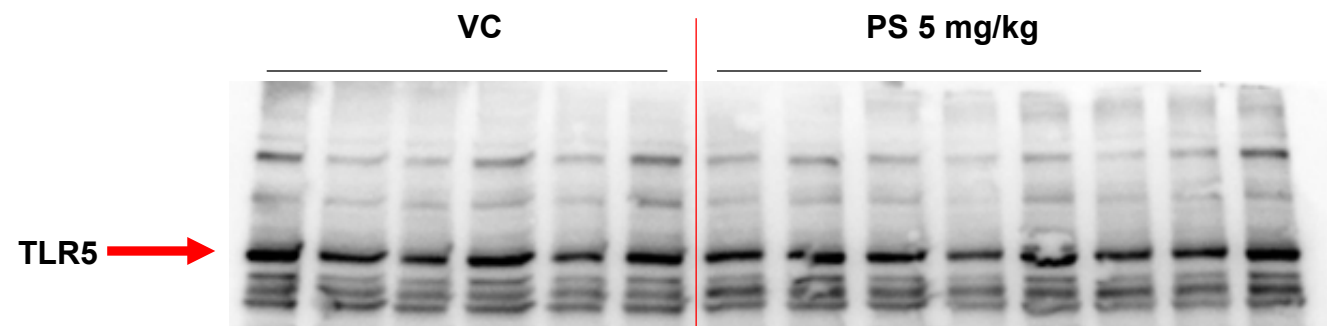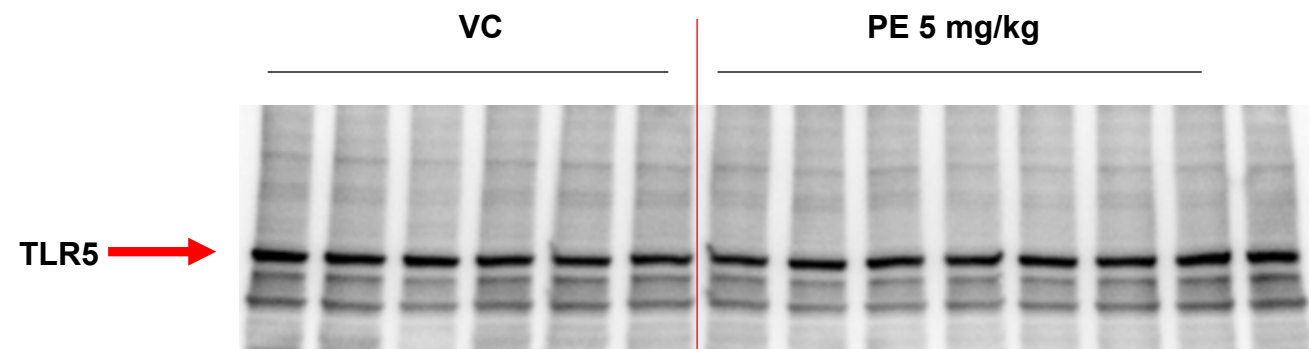

TLR6

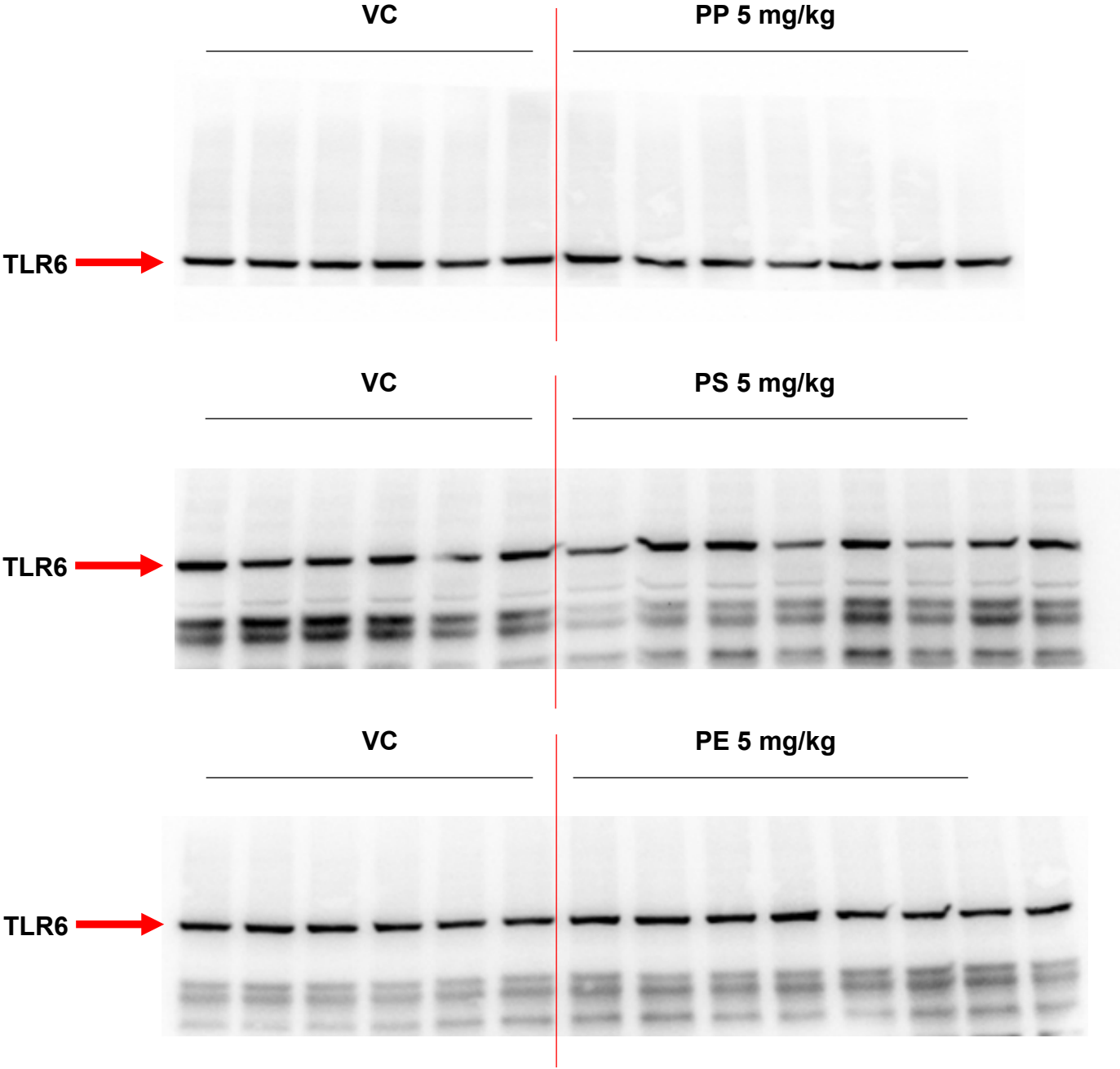

p-I $\kappa$ B- $\alpha$

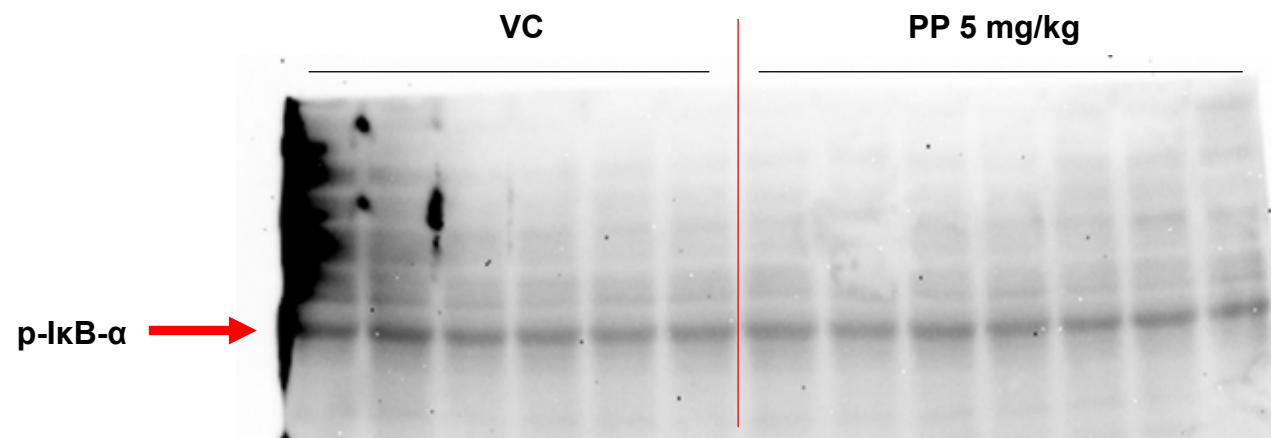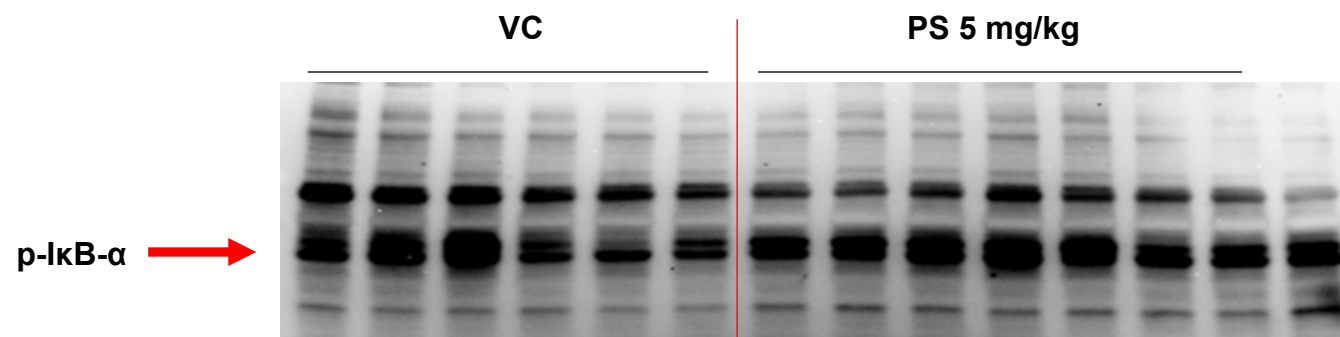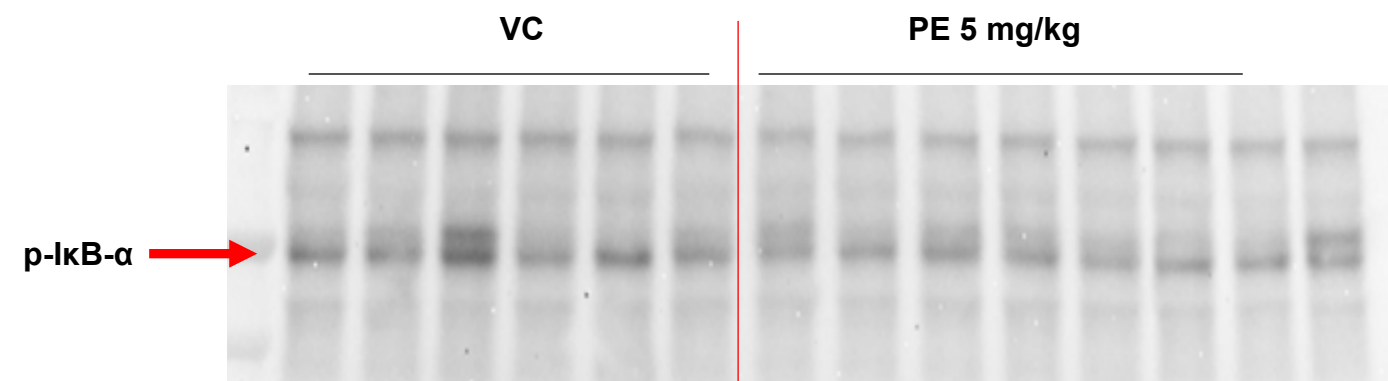

IκB-α

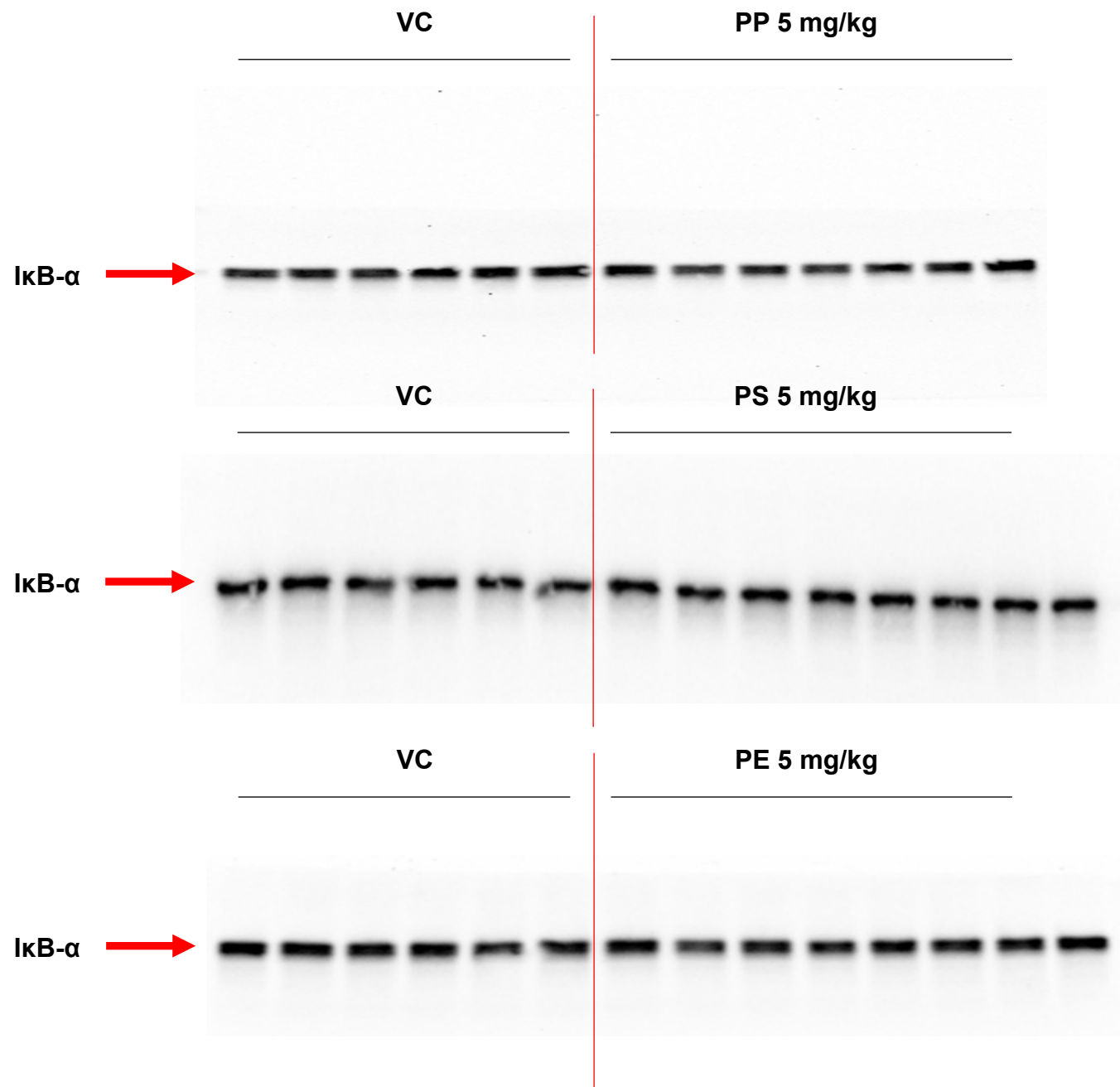

p-NF- $\kappa$ B

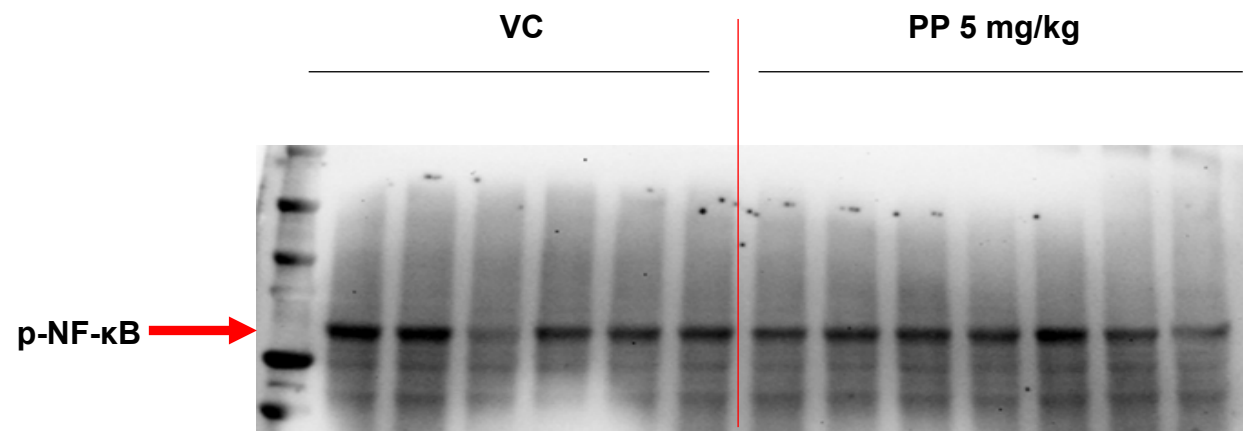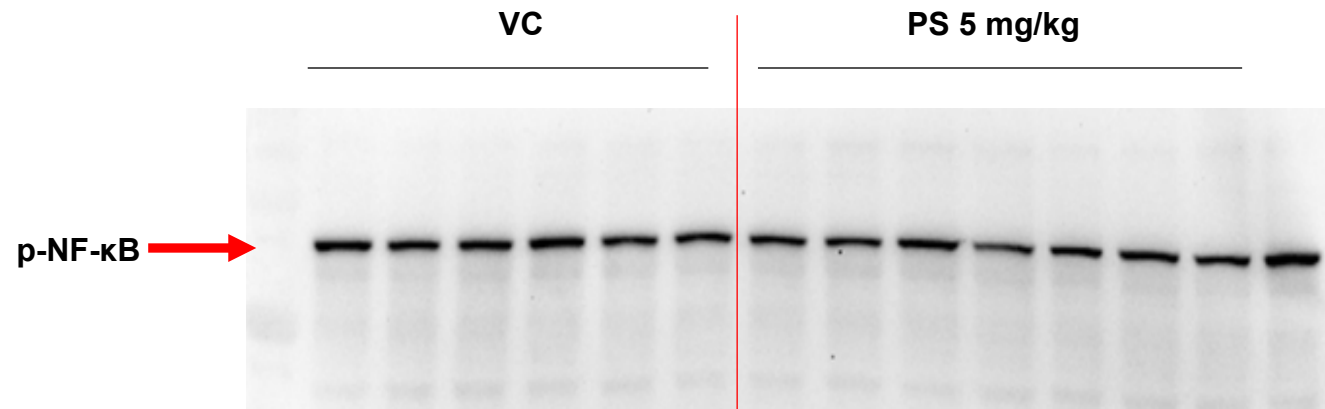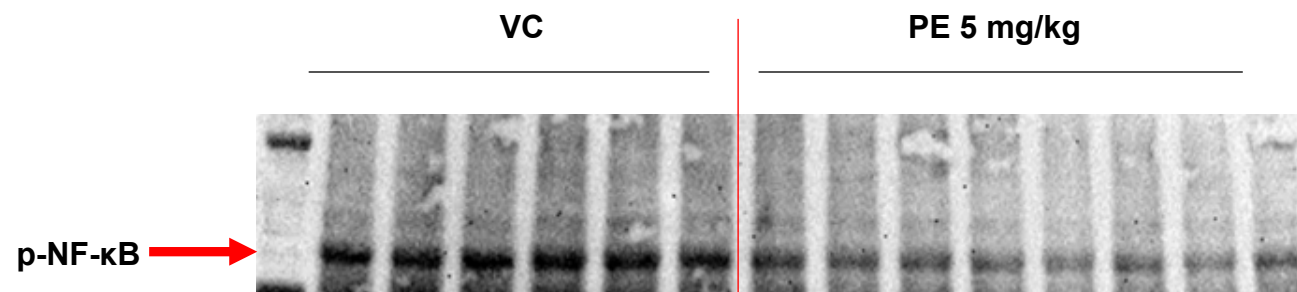

NF- $\kappa$ B

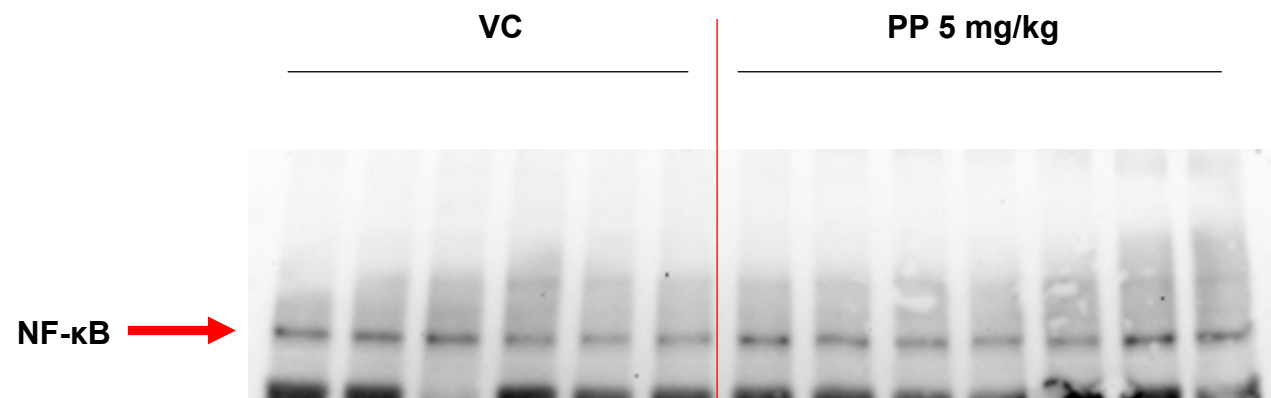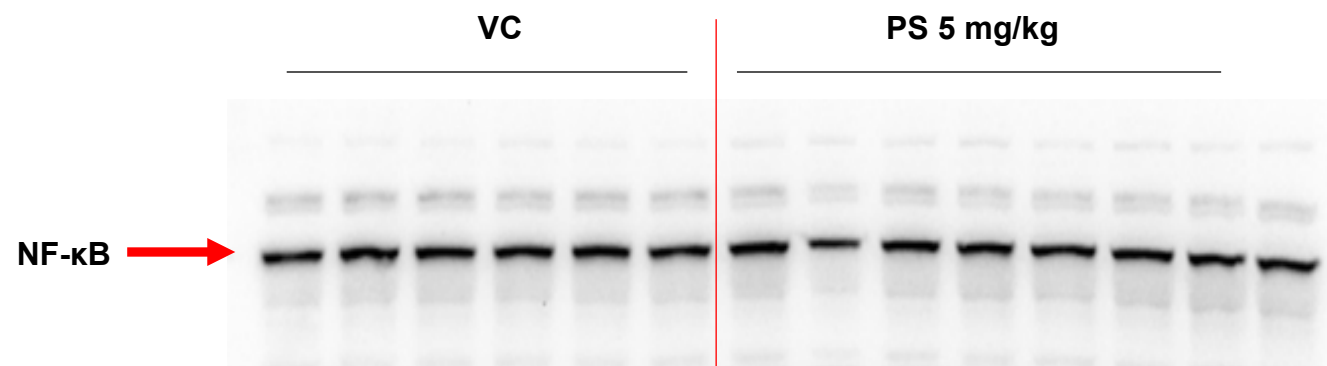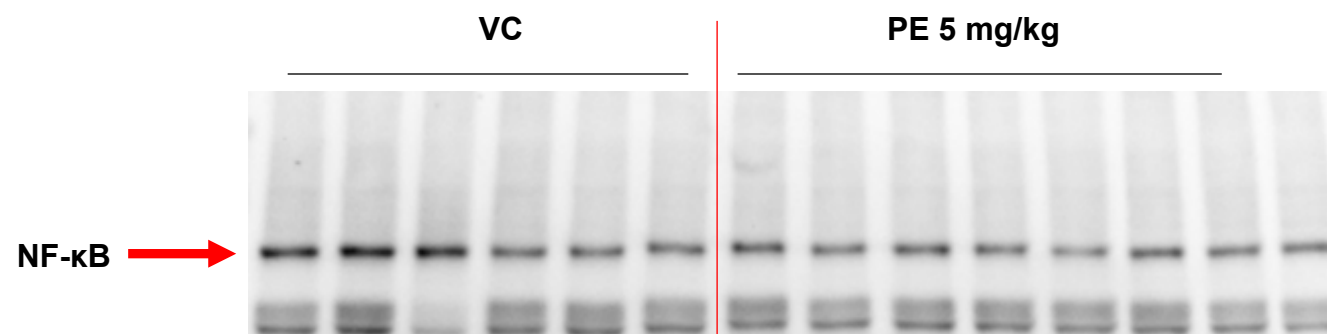

# NLRP3

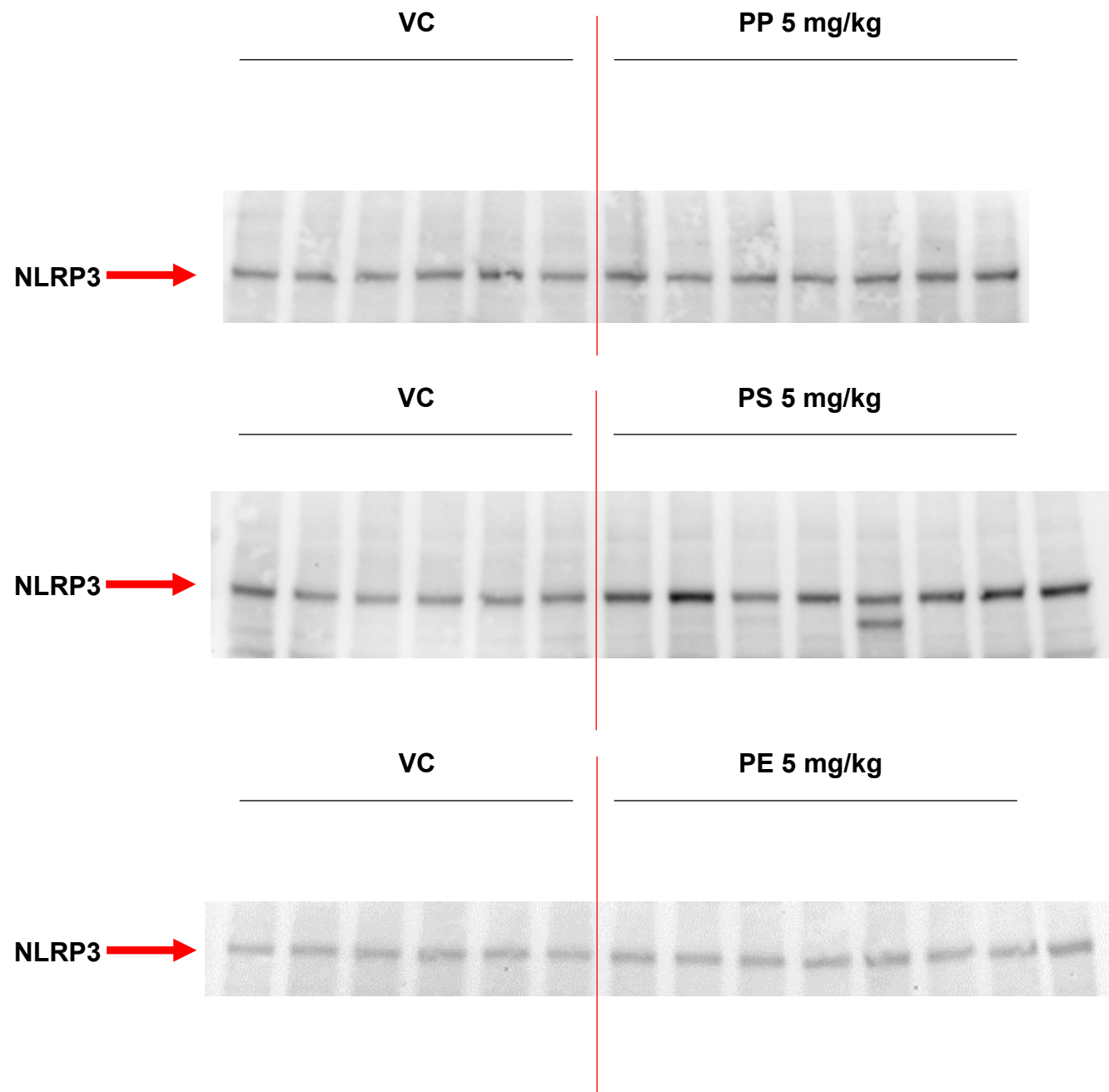

Caspase-1

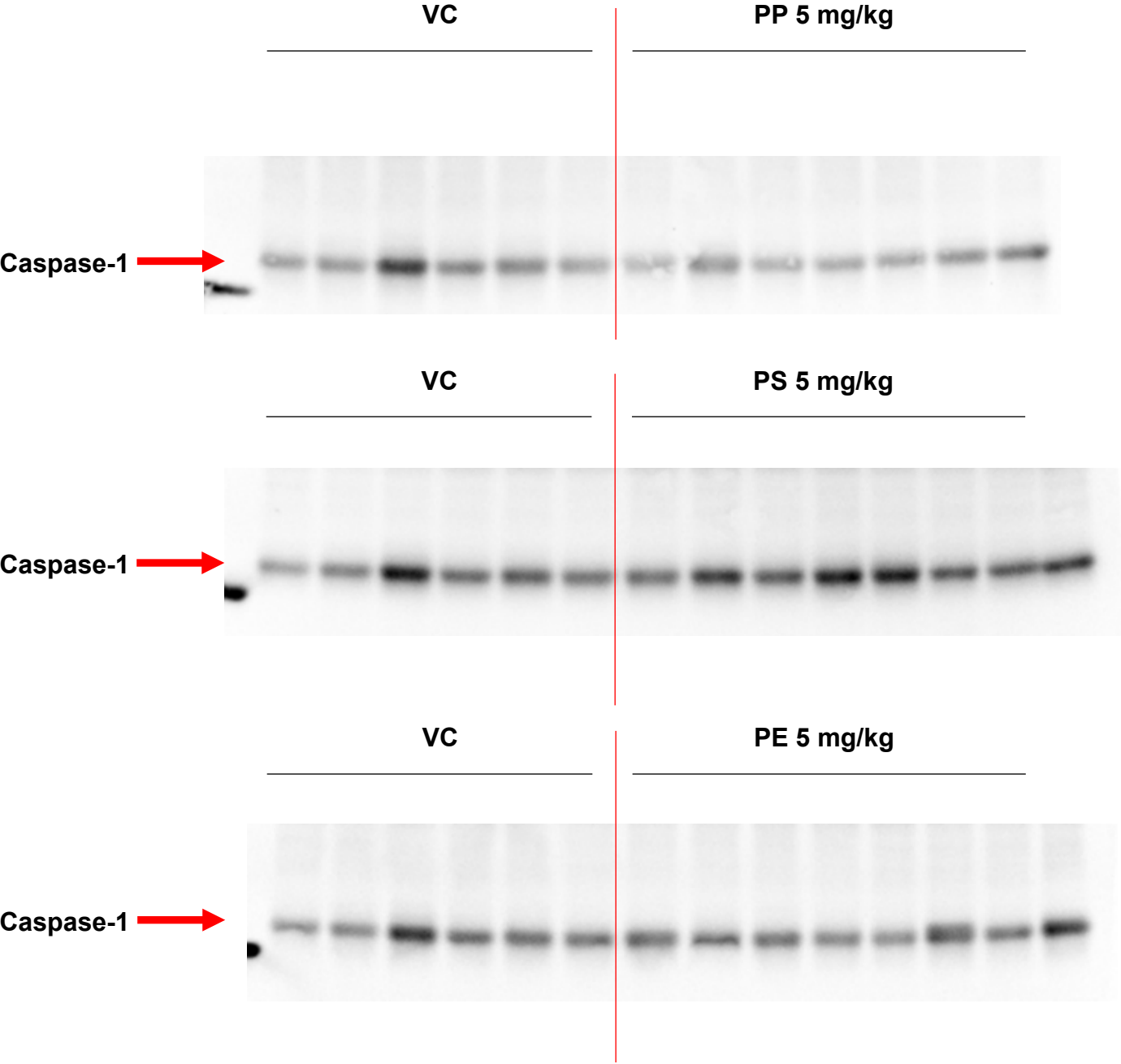

ASC

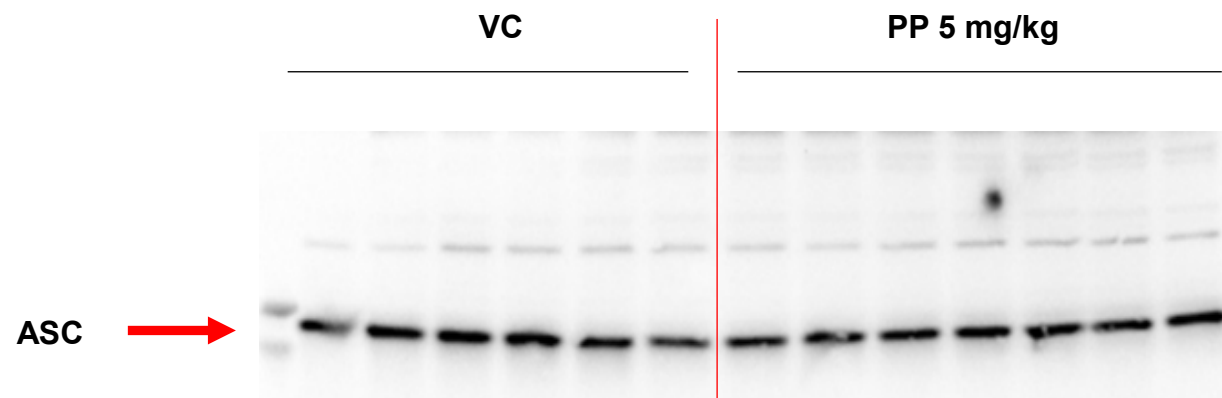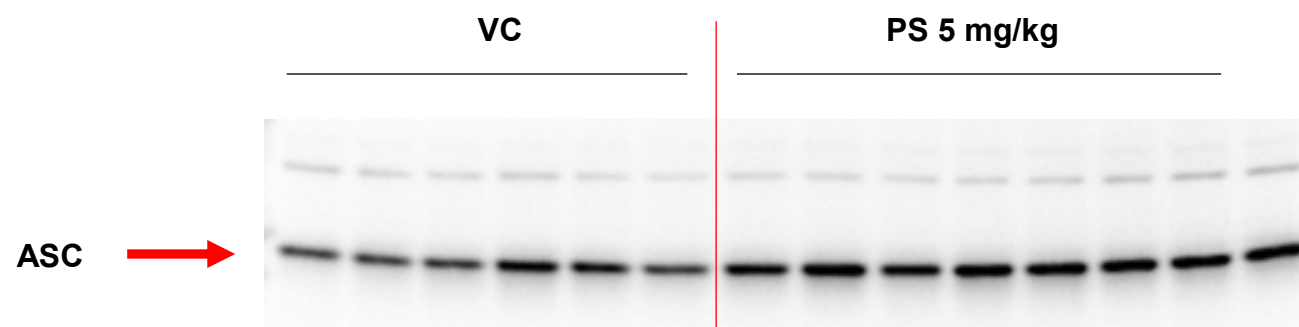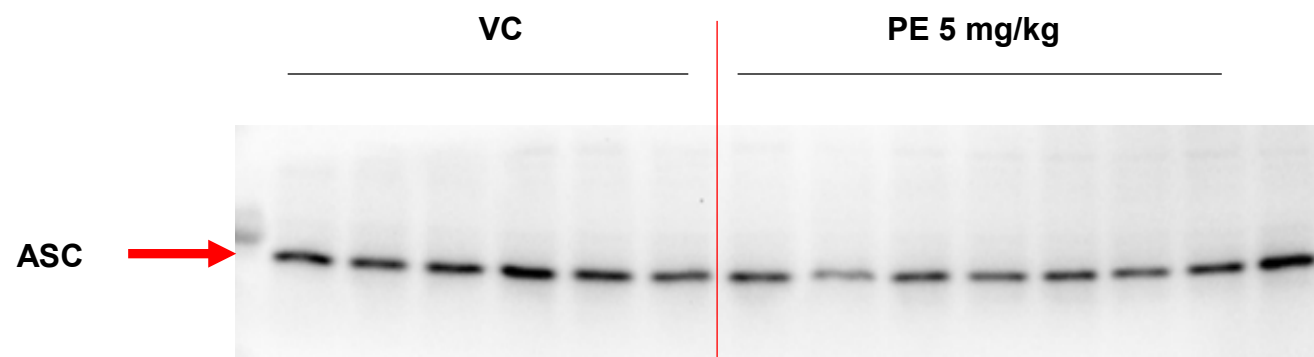

**β-actin**

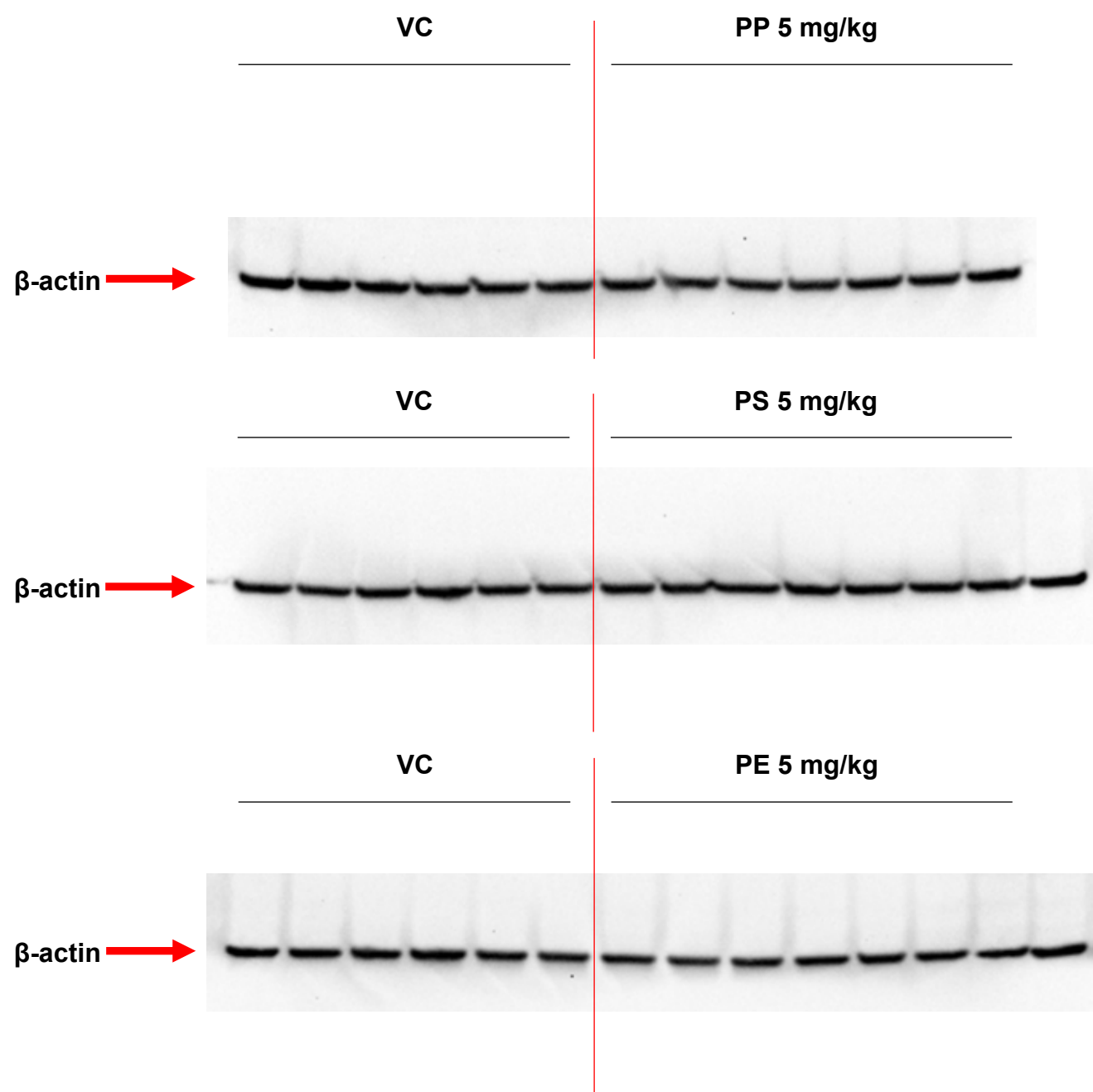

Supplement: Supplementary file 2 — Supplementary file2 (PDF 1728 KB) [file 43188_2023_224_MOESM2_ESM.pdf]
