# Supplementary material for: Pulmonary toxicity assessment of polypropylene, polystyrene, and polyethylene microplastic fragments in mice
Source: Toxicol Res. 2024 Mar 8;40(2):313–23. doi: 10.1007/s43188-023-00224-x (PMC10959865; doi:10.1007/s43188-023-00224-x)
Supplement: Supplementary file 3 — Supplementary file3 (PDF 298 KB) [file 43188_2023_224_MOESM3_ESM.pdf]

editage

Editing Certificate

This document certifies that the manuscript listed below has been edited to ensure language and grammar accuracy and is error free in these aspects. The logical presentation of ideas and the structure of the paper were also checked during the editing process. The edit was performed by professional editors at Editage, a brand of Cactus Communications. The author's core research ideas were not altered in any way during the editing process. The quality of the edit has been guaranteed, with the assumption that our suggested changes have been accepted and the text has not been further altered without the knowledge of our editors.

MANUSCRIPT TITLE

Pulmonary toxicity assessment of polypropylene, polystyrene, and polyethylene microplastic fragments in mice

AUTHORS

JONG-HWAN WOO

ISSUED ON

December 12, 2023

JOB CODE

OZKCC_4_3

THE EDITAGE Promise QUALITY GUARANTEE

Prabh Grewal
Senior Vice President - Editage

editage | helping you get published

Since 2002, Editage has helped over 430,000 authors publish around 1.2 million research papers in scholarly journals across over 1000 disciplines through editorial, translation, transcription, and publication support services. Editage is a brand of Cactus Communications (cactusglobal.com), a science communication and technology company.

CACTUS.

GLOBAL :
+1(833) 979-0061 | request@editage.com

KOREA :
02-3478-4396 | submit-korea@editage.com

editage.com | editage.co.kr | editage.jp | editage.cn | editage.com.br | editage.com.tw | editage.de
